# Supplementary material for: Algorithm for post-clustering curation of DNA amplicon data yields reliable biodiversity estimates
Source: Nat Commun. 2017 Oct 30;8:1188. doi: 10.1038/s41467-017-01312-x (PMC5662604; doi:10.1038/s41467-017-01312-x)
Supplement: Supplementary file 1 — Supplementary Information [file 41467_2017_1312_MOESM1_ESM.pdf]

Supplementary Figures

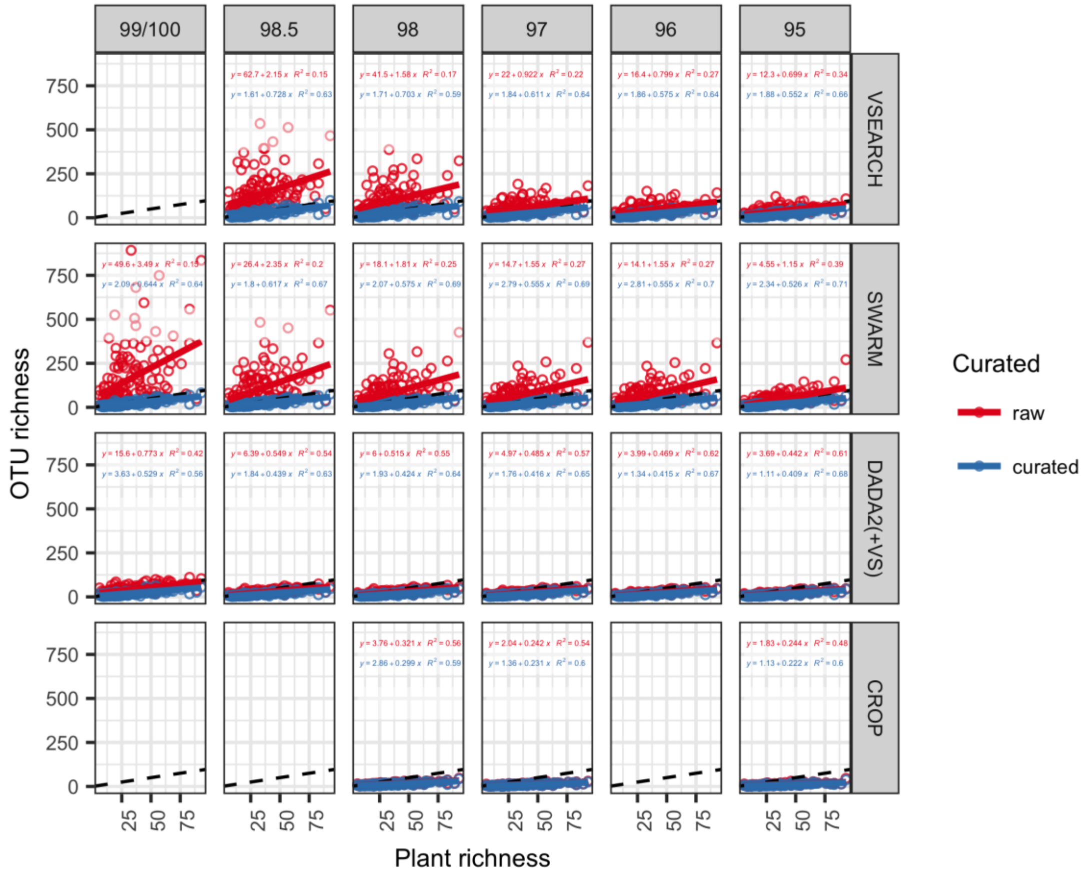

Supplementary Figure 1. Linear regression of OTU richness vs. plant richness.
OTU richness (number of OTUs in each soil sample from the 130 sites) is plotted on the y-axis. Plant richness (number of plant species observed in each of the 130 40m x 40m sites) is plotted on the x-axis. The dashed line is an identity to evaluate whether the OTU count overestimates (to the left of the line) or underestimates (to the right) the plant richness. The linear model for each regression is shown along with the corresponding coefficient of determination (R2). Red points represent the pre-curation methods, and blue point represent post-curation results. The “99/100%” clustering level denotes the pure DADA2 approach (100%) and SWARM with a d-value of 3 (99%). VSEARCH and SWARM initially overestimate the richness significantly at most clustering levels. CROP and DADA2 (+/- clustering) perform better, but all measures of correspondence (R2, slope and intersect) are improved with curation for all methods. The curation effect on CROP is low.

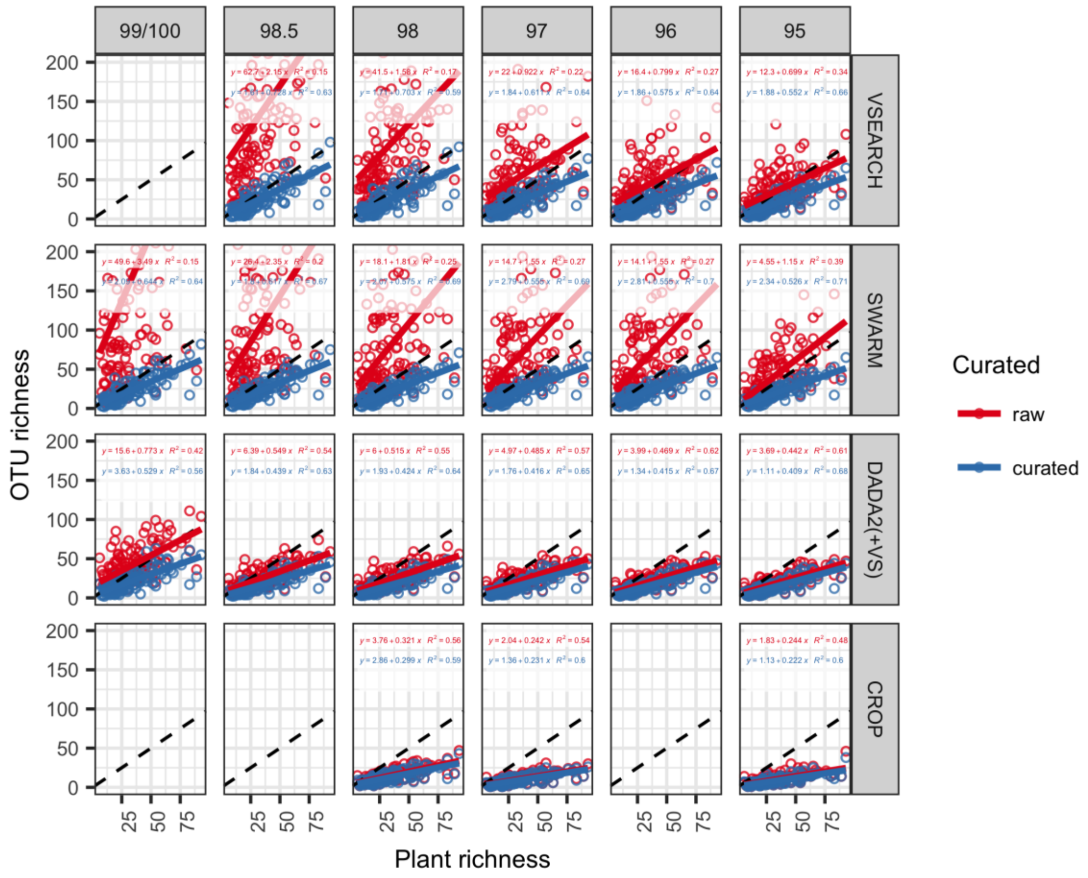

Supplementary Figure 2. Linear regression of OTU richness vs. plant richness.

OTU richness (number of OTUs in each soil sample from the 130 sites) is plotted on the y-axis. Plant richness (number of plant species observed in each of the 130 40m x 40m sites) is plotted on the x-axis. The dashed line is an identity to evaluate whether the OTU count overestimates (to the left of the line) or underestimates (to the right) the plant richness. The linear model for each regression is shown along with the corresponding coefficient of determination (R2). Red points represent the pre-curation methods, and blue point represent post-curation results. The “99/100%” clustering level denotes the pure DADA2 approach (100%) and SWARM with a d-value of 3 (99%). VSEARCH and SWARM initially overestimate the richness significantly at most clustering levels. CROP and DADA2 (+/- clustering) perform better, but all measures of correspondence (R2, slope and intersect) are improved with curation for all methods. The curation effect on CROP is low. Identical to Supplementary Supplementary Figure 1, but shows a truncated y-axis for better illustrattion of the correlations, by excluding the extreme values.

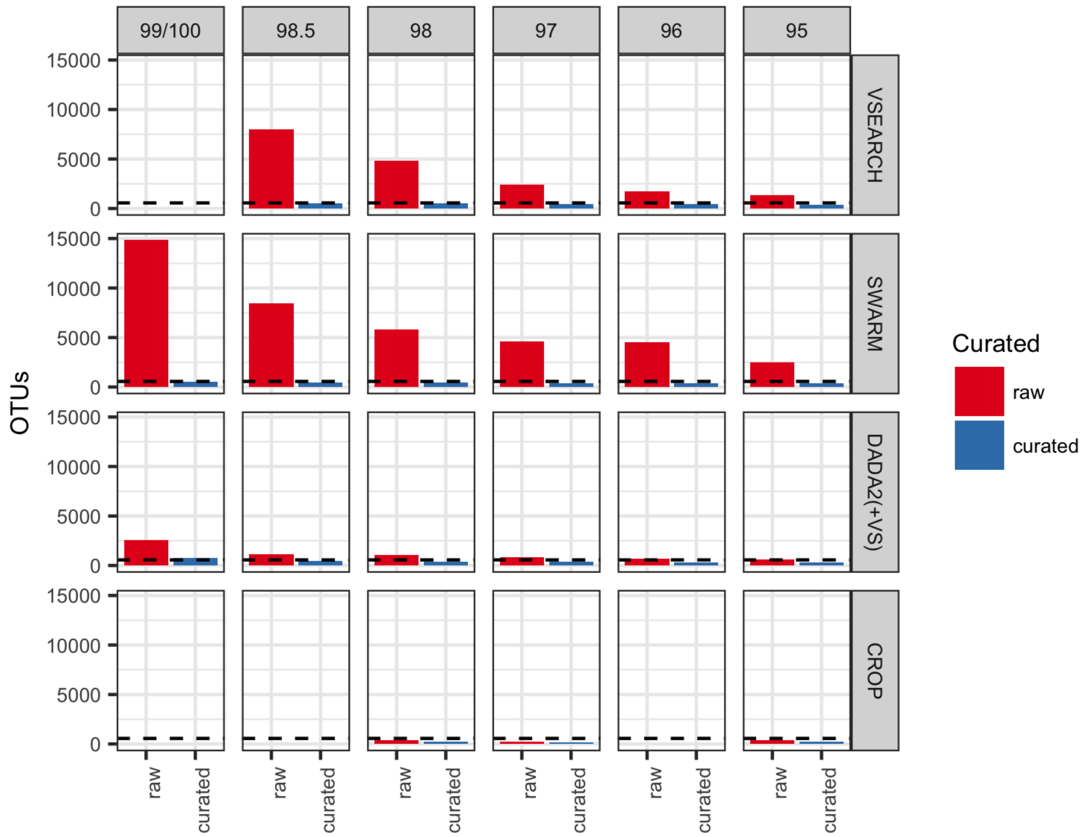

Supplementary Figure 3. Total number of OTUs vs total number of plant species recorded.

Total method level OTU richness is plotted on the y-axis. Red bars represent OTUs of the un-curated methods and blue bars represent OTUs of the curated methods. The dashed line indicates the total number of species (564) observed in the study for comparison. The “99/100%” clustering level denotes the pure DADA2 approach (100%) and SWARM with a d-value of 3 (99%). All methods (except CROP) initially identify many more OTUs than can realistically be expected from the inventories, but all measures of total richness are reduced to realistic levels with curation for all methods. The curation effect on CROP is low.

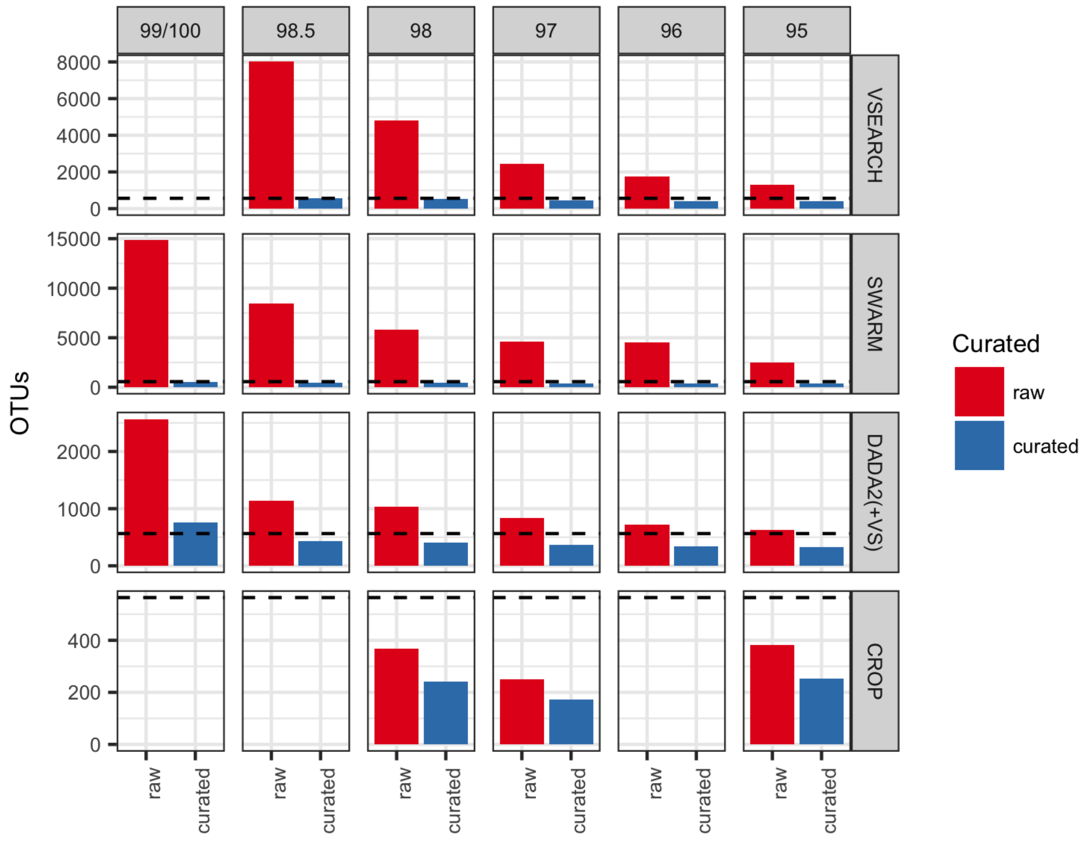

Supplementary Figure 4. Total number of OTUs vs total number of plant species recorded.

Total method level OTU richness is plotted on the y-axis. Red bars represent OTUs of the un-curated methods and blue bars represent OTUs of the curated methods. The dashed line indicates the total number of species (564) observed in the study for comparison. The “99/100%” clustering level denotes the pure DADA2 approach (100%) and SWARM with a d-value of 3 (99%). All methods (except CROP) initially identify many more OTUs than can realistically be expected from the inventories, but all measures of total richness are reduced to realistic levels with curation for all methods. The curation effect on CROP is low. Identical to Supplementary Figure 3, but with a flexible y-axis for better comparison of the low richness CROP method.

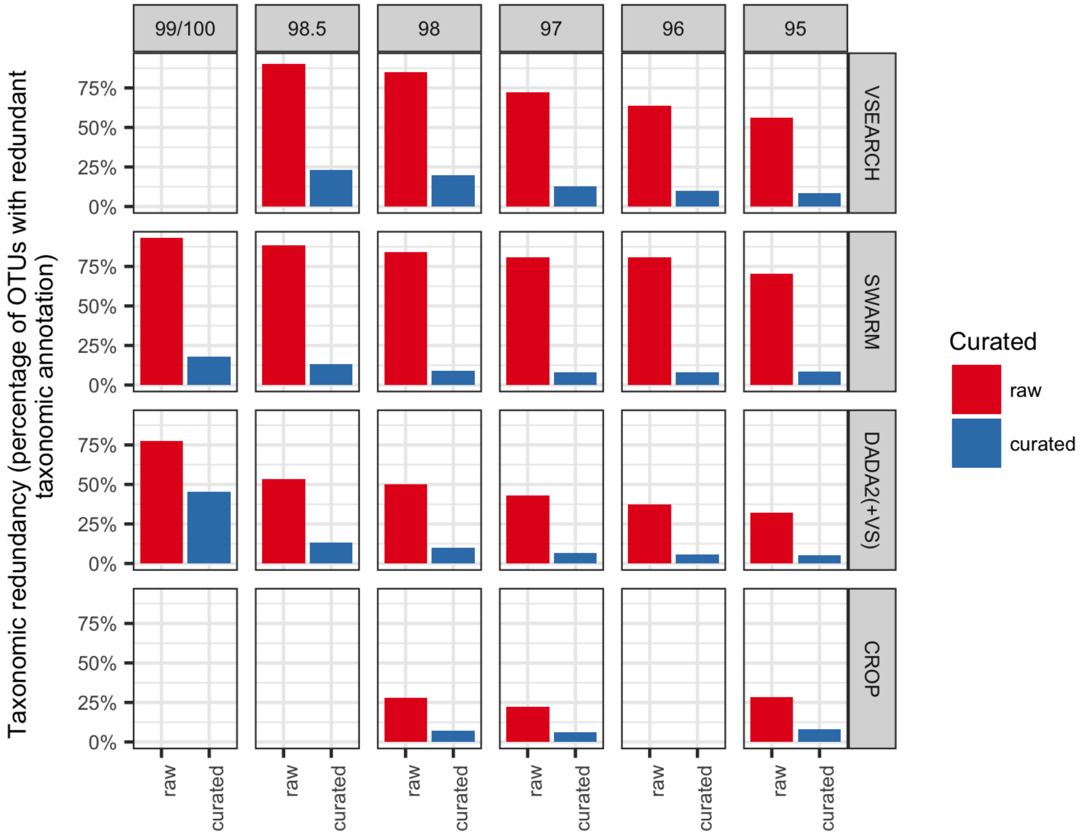

Supplementary Figure 5. Taxonomic redundancy of each method. Taxonomic redundancy (the proportion of OTUs with a redundant taxonomic assignment) is plotted on the y-axis. Red bars represent the redundancy of the taxonomic assignment from the un-curated methods and blue bars represent that of the curated methods. The “99/100%” clustering level denotes the pure DADA2 approach (100%) and SWARM with a d-value of 3 (99%). All methods initially have a high taxonomic redundancy – even CROP, which contains very few OTUs – but taxonomic redundancy is reduced to realistic levels with curation for all methods.

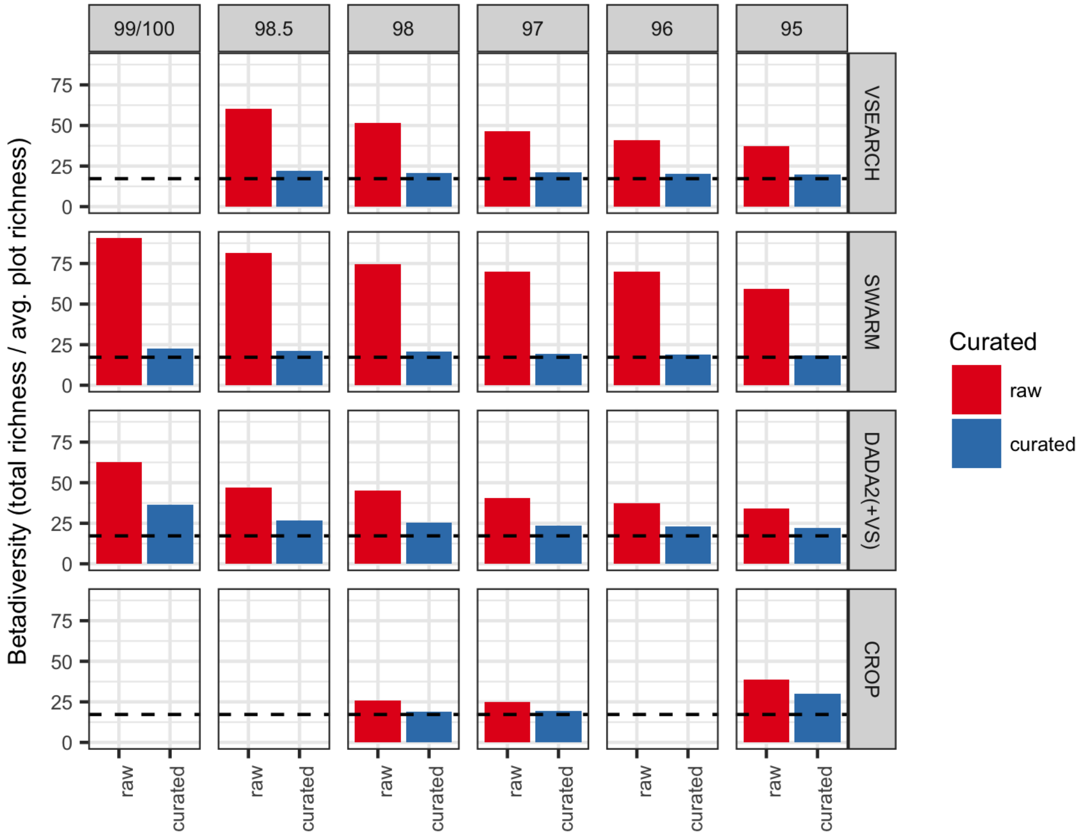

Supplementary Figure 6. Betadiversity of each method.

Betadiversity (calculated as total number of OTUs divided by the mean number of OTUs in the 130 sites) is plotted on the y-axis. Red bars represent the betadiversity of the taxonomic assignment from the un-curated methods and blue bars represent that of the curated methods. The dashed line indicates the betadiversity of the plant data (17.23) observed in the study for comparison, calculated in the same way. The “99/100%” clustering level denotes the pure DADA2 approach (100%) and SWARM with a d-value of 3 (99%). All methods initially have a higher betadiversity than can be expected from observational plant data – even CROP, which contain very few OTU. The betadiversity is reduced to realistic levels with curation for all methods.

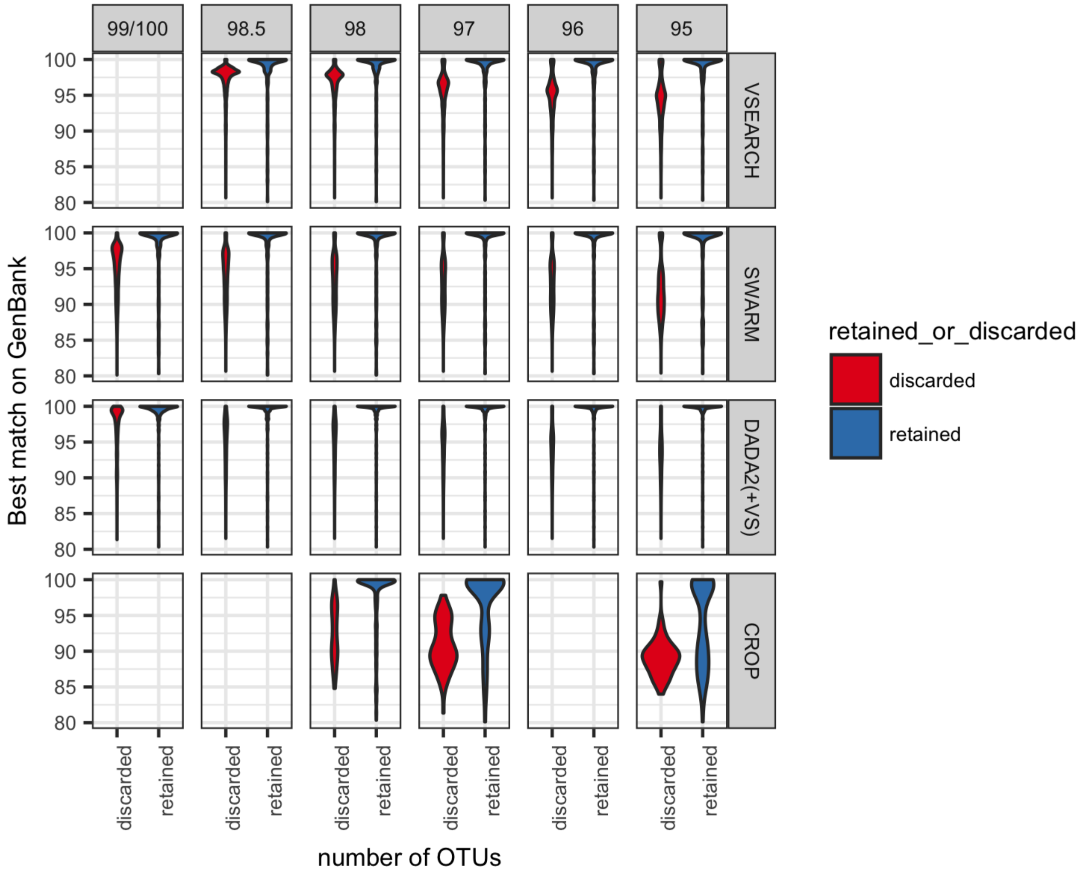

Supplementary Figure 7. Distribution of best matches for OTUs on GenBank.

Density distribution of the best reference database match for all OTUs (percent identity (%) of best matching reference sequence on GenBank) is plotted as a violin plot. Red bars represent OTUs discarded by the LULU algorithm, and blue bars represent OTUs retained/curated by the algorithm. The “99/100%” clustering level denotes the pure DADA2 approach (100%) and SWARM with a d-value of 3 (99%). For all methods the curation of LULU discards primarily OTUs with best matches of less than 99-100%, and retains predominantly perfectly or near-perfectly matching sequences. The CROP method contains a much higher proportion of OTUs with low matches, even after curation.

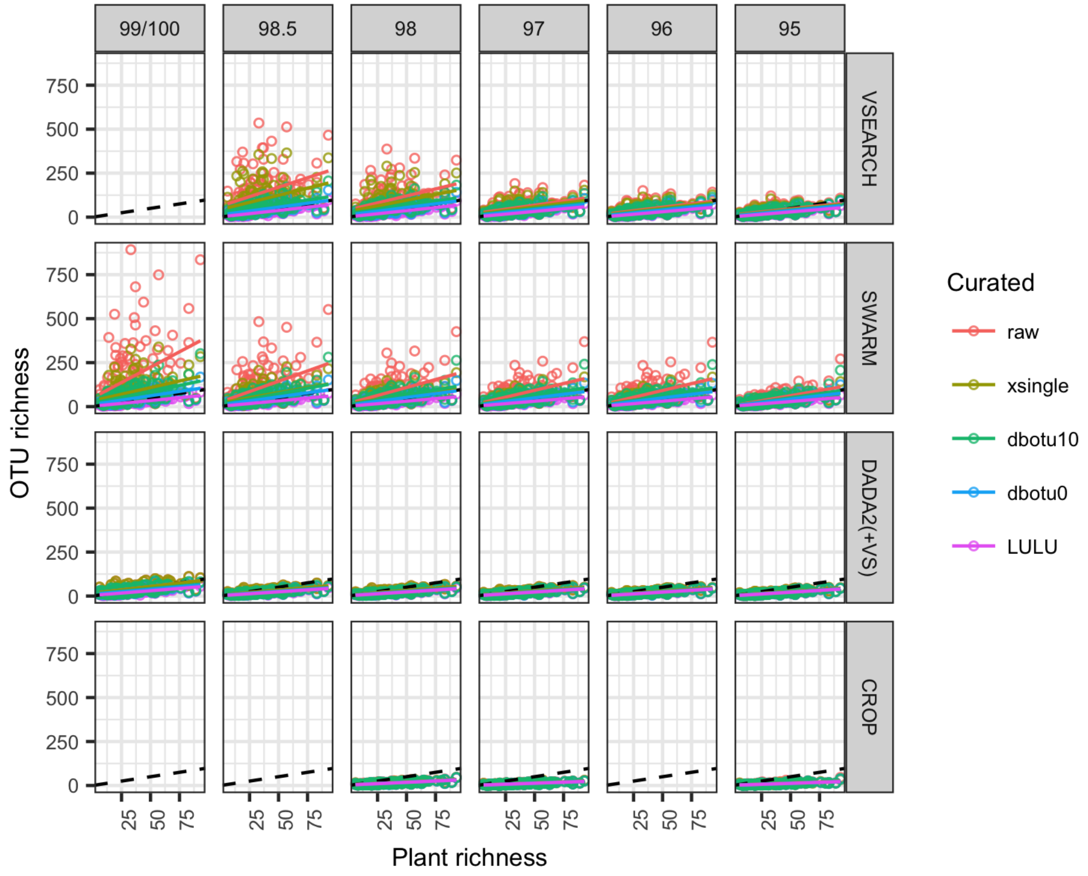

Supplementary Figure 8. OTU richness vs. plant richness - LULU curation, singleton culling, curation with dbotu3.

OTU richness (number of OTUs in each soil sample from the 130 sites) is plotted on the y-axis. Plant richness (number of plant species observed in each of the 130 40m x 40m sites) is plotted on the x-axis. Values are shown for un-curated OTU tables, tables with singletons removed (xsingle), tables curated with dbotu3, abundance criterion 10 (dbotu10), tables curated with dbotu3, abundance criterion 0 (dbotu0) and tables curated with LULU. The dashed line is an identity to evaluate whether the OTU count overestimates (to the left of the line) or underestimates (to the right) the plant richness. The “99/100%” clustering level denotes the pure DADA2 approach (100%) and SWARM with a d-value of 3 (99%). (a) shows the full y-axis, whereas (b) shows a truncated y-axis to better illustrate the correlations, by excluding the extreme values. Statistics of the regression can be seen in Supplementary Table 3. Although difficult to see from the plots, correspondence with plant data was best for the LULU curation.

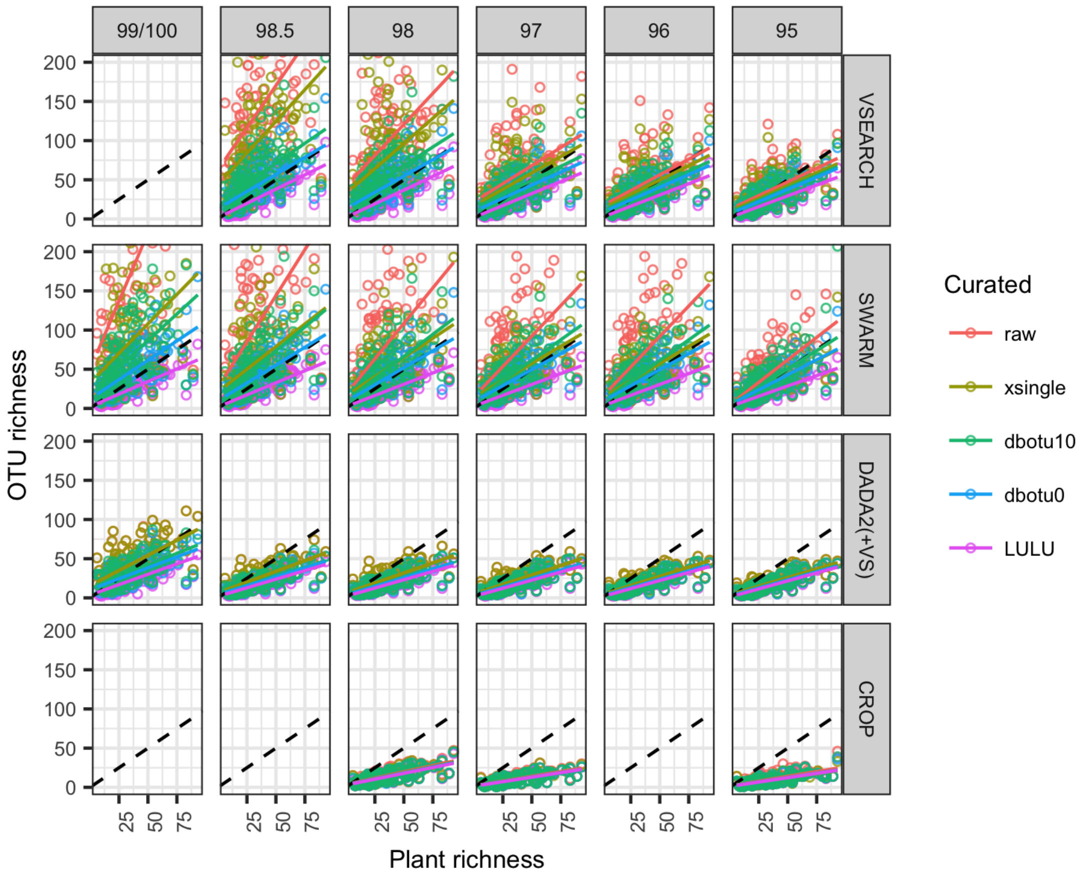

Supplementary Figure 9. OTU richness vs. plant richness - LULU curation, singleton culling, curation with dbotu3.

OTU richness (number of OTUs in each soil sample from the 130 sites) is plotted on the y-axis. Plant richness (number of plant species observed in each of the 130 40m x 40m sites) is plotted on the x-axis. Values are shown for un-curated OTU tables, tables with singletons removed (xsingle), tables curated with dbotu3, abundance criterion 10 (dbotu10), tables curated with dbotu3, abundance criterion 0 (dbotu0) and tables curated with LULU. The dashed line is an identity to evaluate whether the OTU count overestimates (to the left of the line) or underestimates (to the right) the plant richness. The "99/100%" clustering level denotes the pure DADA2 approach (100%) and SWARM with a d-value of 3 (99%). Statistics of the regression can be seen in Supplementary Table 3. Although difficult to see from the plots, correspondence with plant data was best for the LULU curation. Identical to Supplementary Figure 8, but with a truncated y-axis to better illustrate the correlations, by excluding the extreme values.

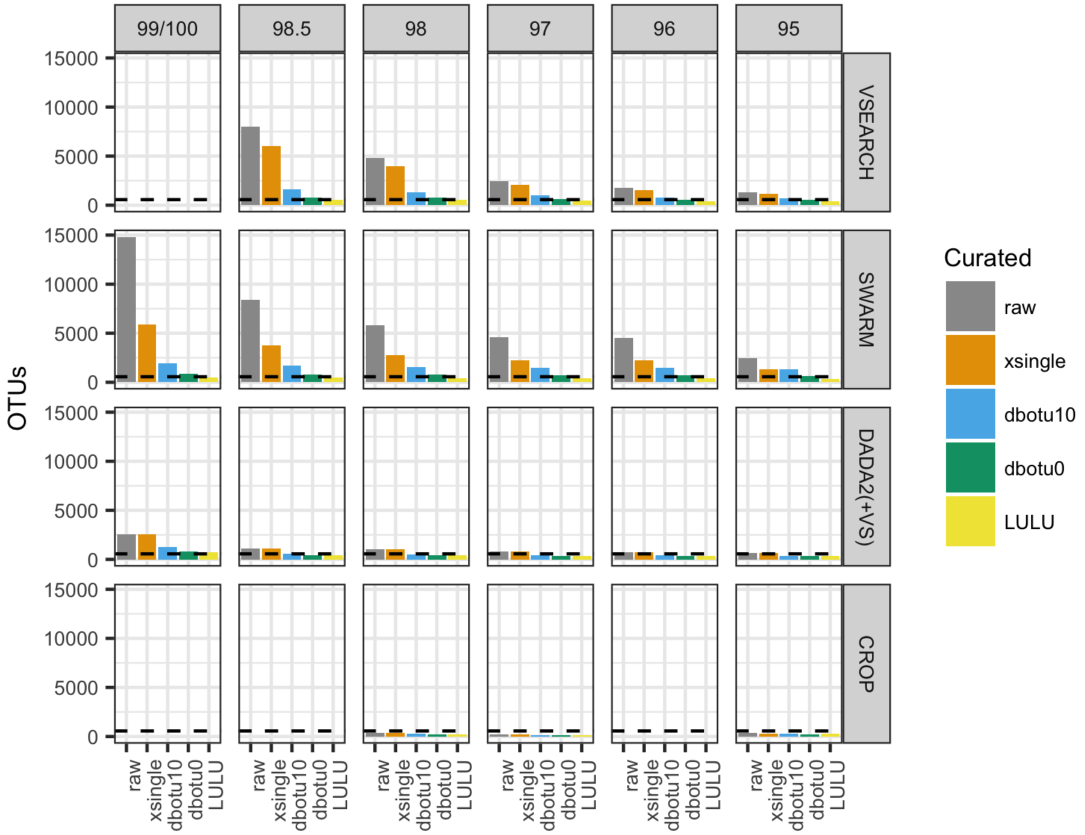

Supplementary Figure 10. Total number of OTUs - LULU curation, singleton culling, curation with dbotu3.

Total method level OTU richness is plotted on the y-axis. Values are shown for un-curated OTU tables, tables with singletons removed (xsingle), tables curated with dbotu3, abundance criterion 10 (dbotu10), tables curated with dbotu3, abundance criterion 0 (dbotu0) and tables curated with LULU. The dashed line indicates the total number of species (564) observed in the study for comparison. LULU consistently performed better on tables from the two greedy algorithms, but dbotu3 resulted in a comparable curation for the other approaches.

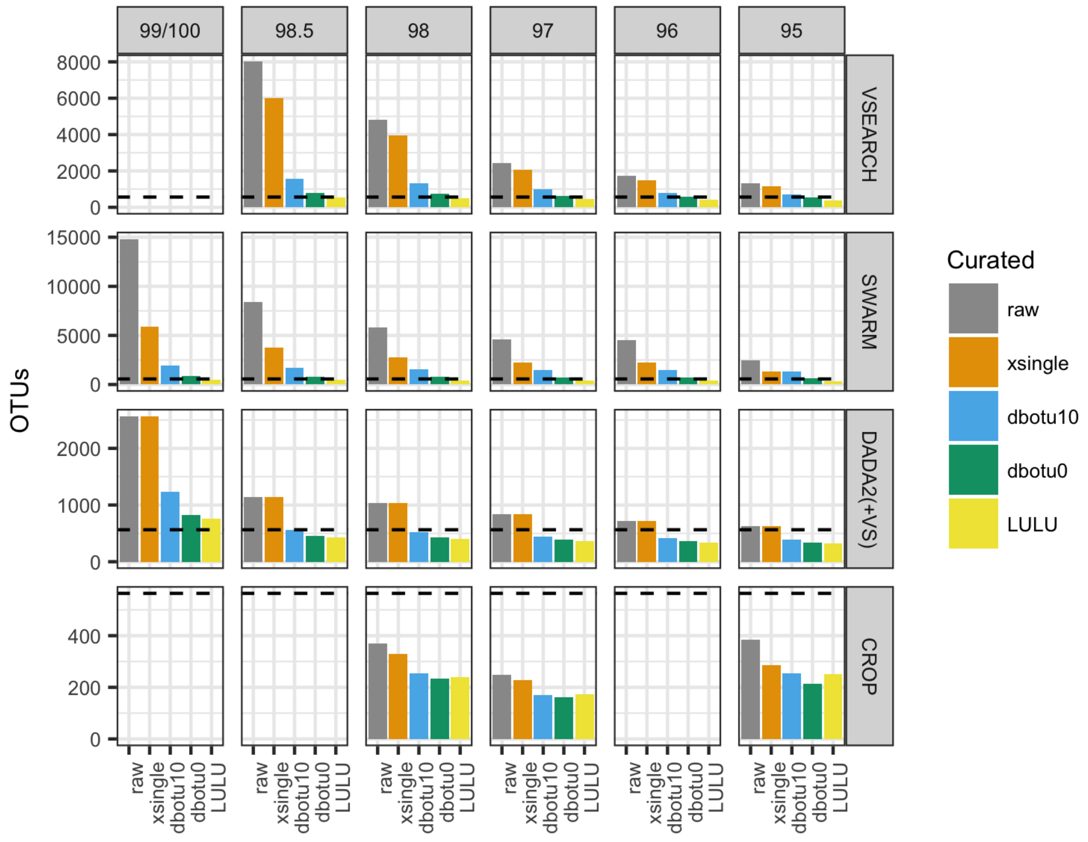

Supplementary Figure 11. Total number of OTUs - LULU curation, singleton culling, curation with dbotu3.

Total method level OTU richness is plotted on the y-axis. Values are shown for un-curated OTU tables, tables with singletons removed (xsingle), tables curated with dbotu3, abundance criterion 10 (dbotu10), tables curated with dbotu3, abundance criterion 0 (dbotu0) and tables curated with LULU. The dashed line indicates the total number of species (564) observed in the study for comparison. The “99/100%” clustering level denotes the pure DADA2 approach (100%) and SWARM with a d-value of 3 (99%). LULU consistently performed better on tables from the two greedy algorithms, but dbotu3 resulted in a comparable curation for the other approaches. Identical to Supplementary Figure 10, but with a flexible y-axis for better comparison of the low richness CROP method.

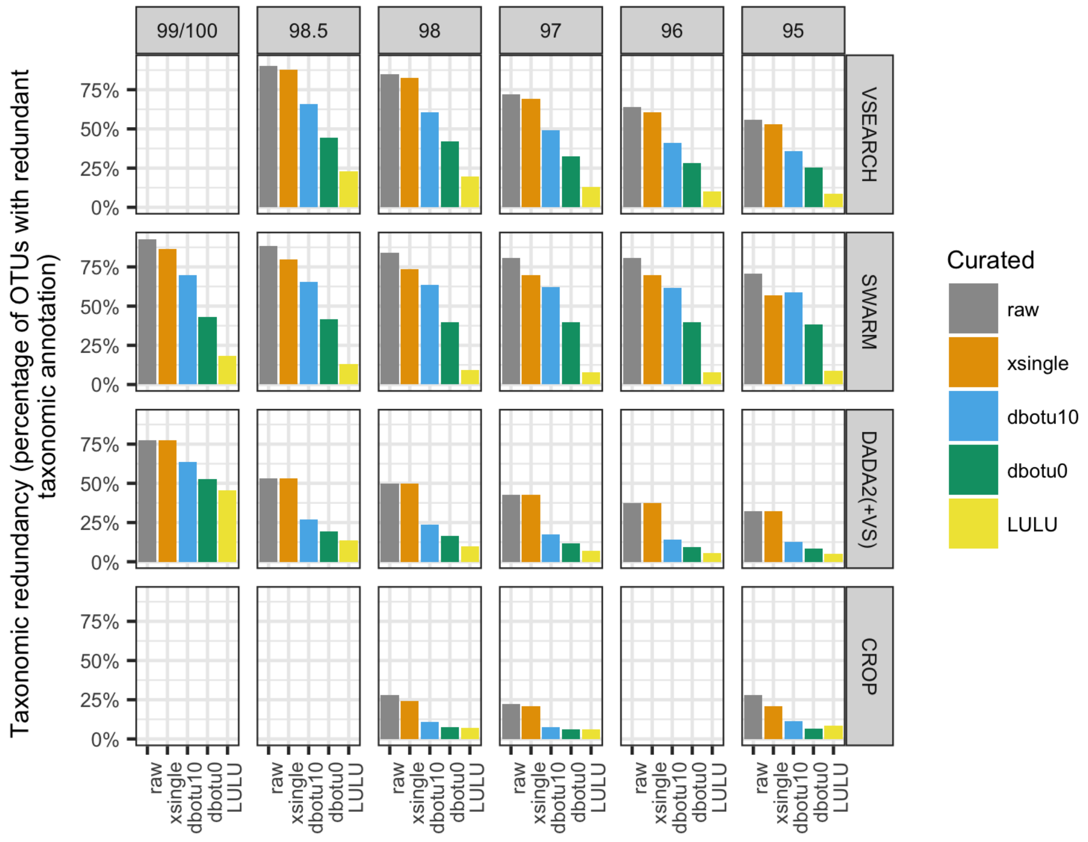

Supplementary Figure 12. Taxonomic redundancy - LULU curation, singleton culling, curation with dbotu3.

Taxonomic redundancy (the proportion of OTUs with a redundant taxonomic assignment) is plotted on the y-axis. Values are shown for un-curated OTU tables, tables with singletons removed (xsingle), tables curated with dbotu3, abundance criterion 10 (dbotu10), tables curated with dbotu3, abundance criterion 0 (dbotu0) and tables curated with LULU. The “99/100%” clustering level denotes the pure DADA2 approach (100%) and SWARM with a d-value of 3 (99%). LULU performed best in all comparisons.

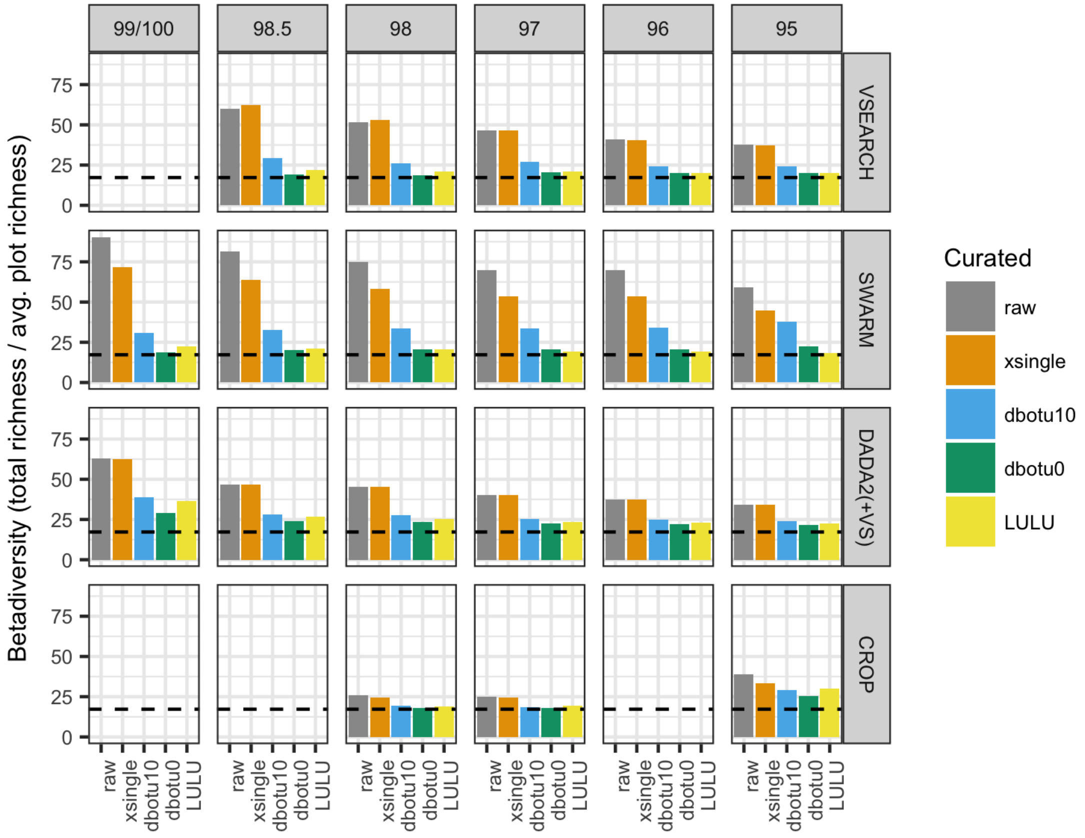

Supplementary Figure 13. Betadiversity - LULU curation, singleton culling, curation with dbotu3.

Betadiversity (calculated as total number of OTUs divided by the mean number of OTUs in the 130 sites) is plotted on the y-axis. Values are shown for un-curated OTU tables, tables with singletons removed (xsingle), tables curated with dbotu3, abundance criterion 10 (dbotu10), tables curated with dbotu3, abundance criterion 0 (dbotu0) and tables curated with LULU. The dashed line indicates the betadiversity of the plant data (17.23) observed in the study for comparison, calculated in the same way. The “99/100%” clustering level denotes the pure DADA2 approach (100%) and SWARM with a d-value of 3 (99%). All approaches result in more realistic betadiversity estimates, but LULU and dbotu3 (a=0) consistently performed best.

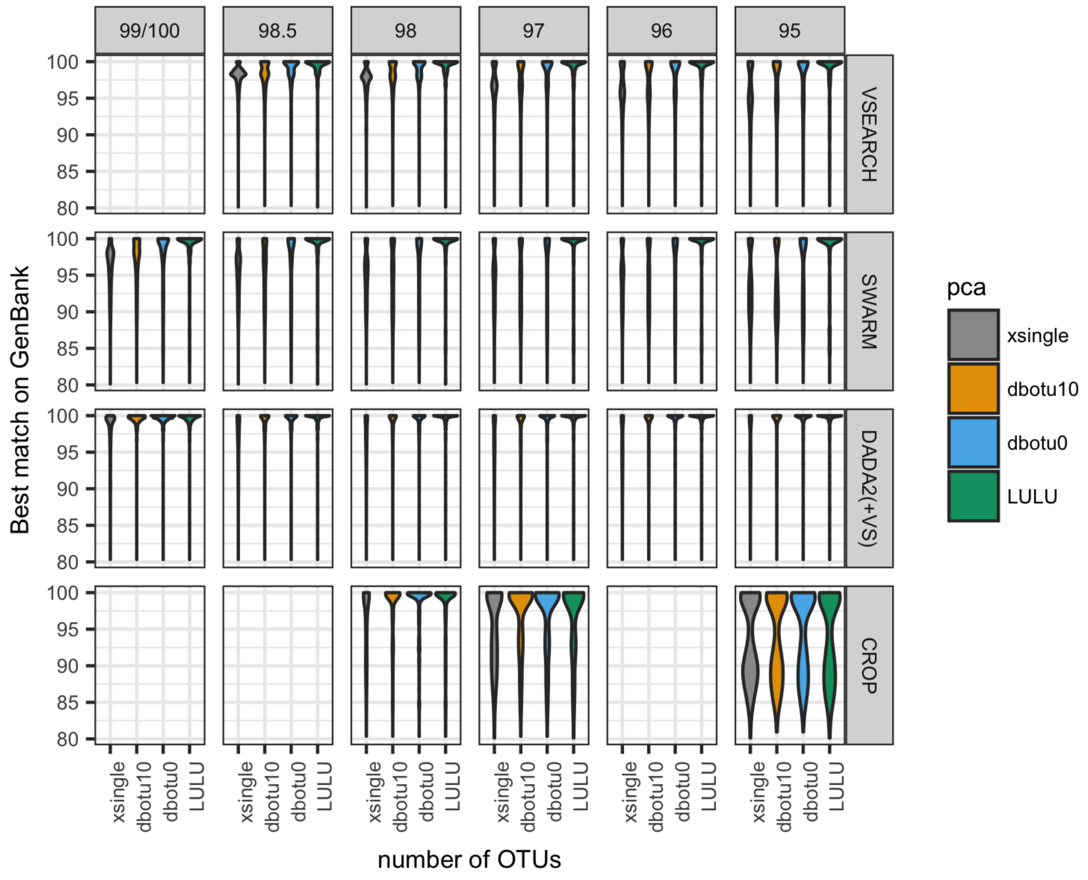

Supplementary Figure 14. Distribution of best matches of retained OTUs - LULU curation, singleton culling, curation with dbotu3.

Density distribution of the best reference database match for all retained OTUs (percent identity (%) of best matching reference sequence on GenBank) is plotted as a violin plot. Values are shown for OTU tables with singletons removed, tables processed with dbotu3 (a=10), and tables processed with dbotu3 (a=10) and tables curated with LULU. The “99/100%” clustering level denotes the pure DADA2 approach (100%) and SWARM with a d-value of 3 (99%). Although distributions are remarkably similar, some of the distribution show that LULU retains a higher proportion of perfect matches.

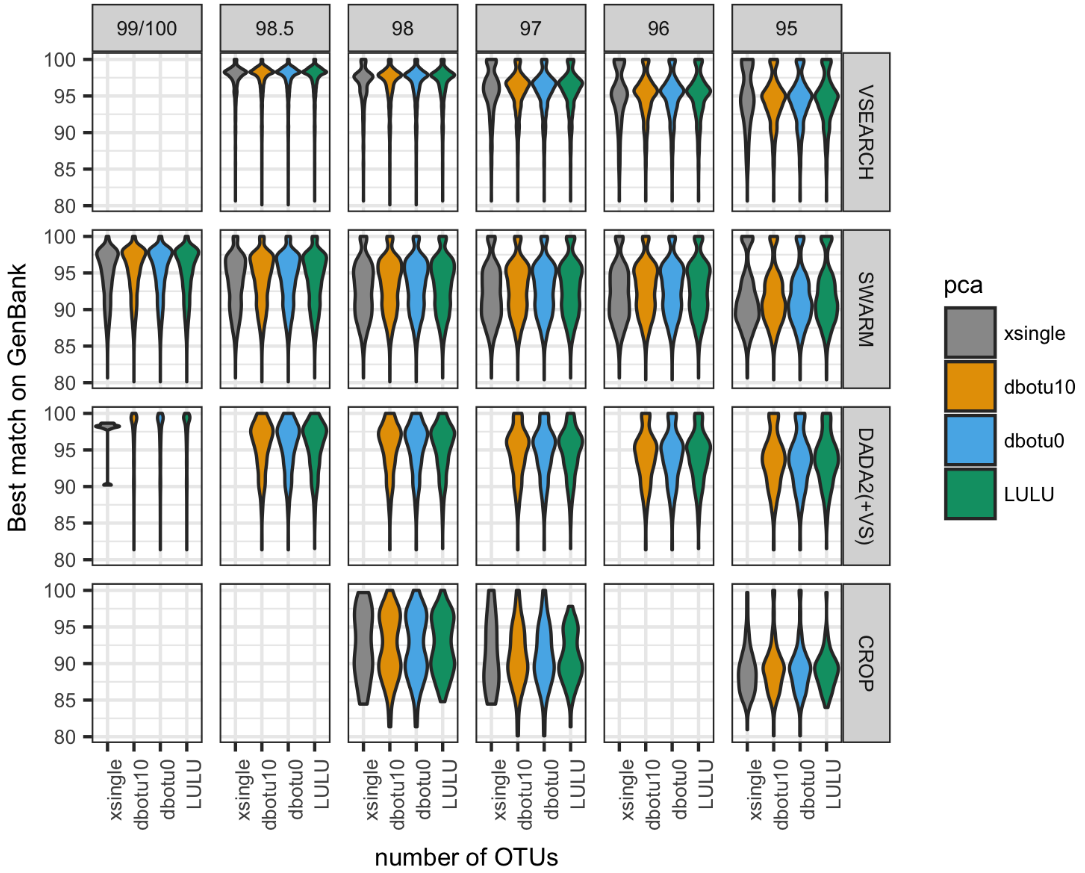

Supplementary Figure 15. Distribution of best matches of discarded OTUs - LULU curation, singleton culling, curation with dbotu3.

Density distribution of the best reference database match for all discarded OTUs (percent identity (%) of best matching reference sequence on GenBank) is plotted as a violin plot. Values are shown for OTU tables with singletons removed, tables processed with dbotu3 (a=10), and tables processed with dbotu3 (a=10) and tables curated with LULU. The “99/100%” clustering level denotes the pure DADA2 approach (100%) and SWARM with a d-value of 3 (99%). Distributions are very similar, and no clear differences can be seen.

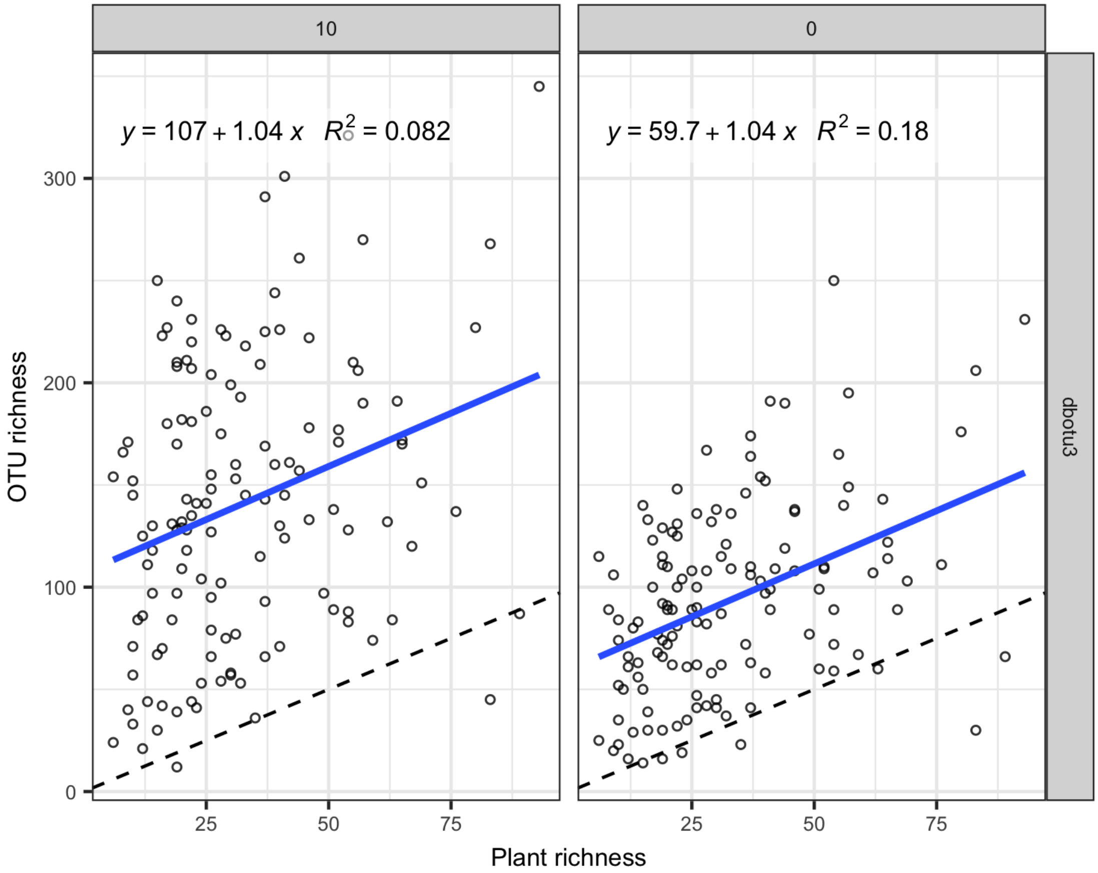

Supplementary Figure 16. OTU richness vs. plant richness - dbotu3 as a 'one-step' clustering tool.

OTU richness (number of OTUs in each soil sample from the 130 sites) is plotted on the y-axis. Plant richness (number of plant species observed in each of the 130 40m x 40m sites) is plotted on the x-axis. Values are shown for the two OTU tables produced with the dbotu3 algorithm as a one-step tool with two different abundance cutoff settings: abundance criterion 10 (intended for removing sequencing errors) and 0 (aimed at accounting for only sequencing error). The dashed line is an identity to evaluate whether the OTU count overestimates (to the left of the line) or underestimates (to the right) the plant richness. Statistics of the regression can be seen in the top.

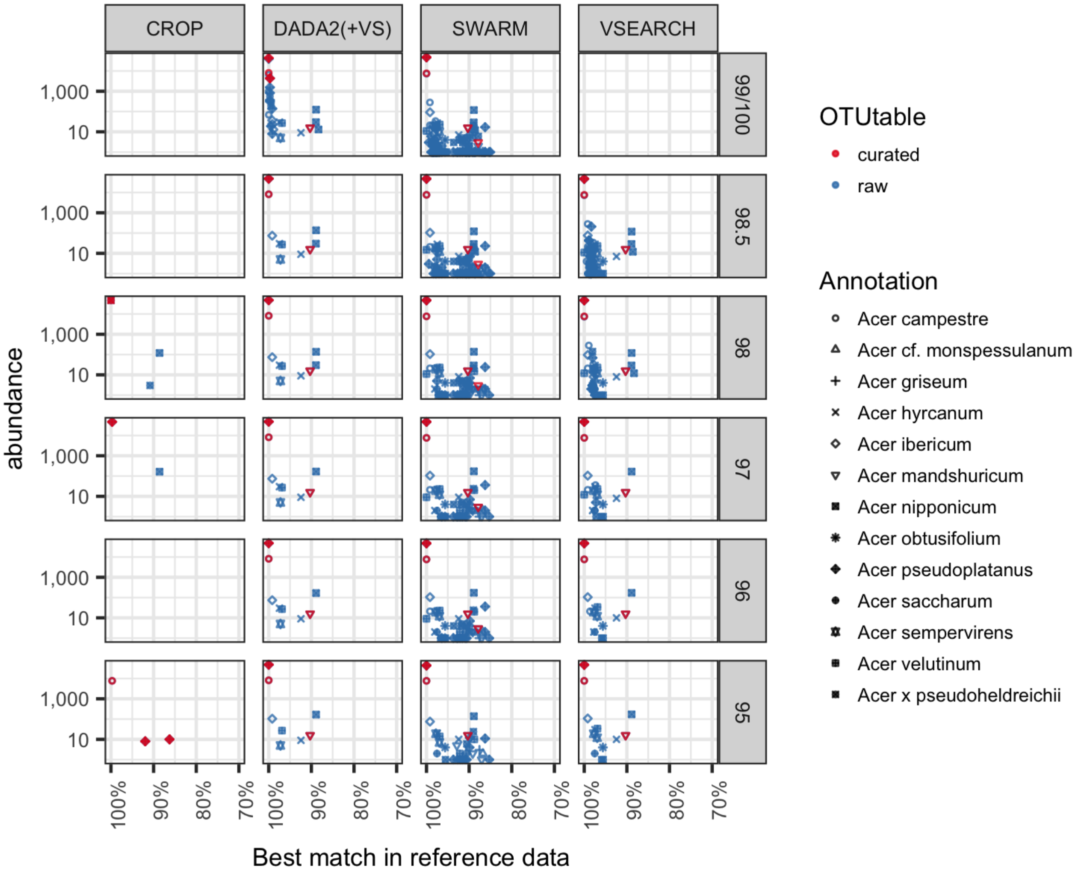

Supplementary Figure 17. Curation effect on OTUs assigned to *Acer*. Three species of *Acer* (*A. campestre, A. pseudoplatanus* and *A. platanoides*) were recorded in the plant survey. Curation resulted in accurate diversity measures (except for CROP) and the annotation was also correct for two of the species. The third OTU imperfectly assigned to *Acer mandshuricum* probably indicates that no perfect match for the third species (*A. platanoides*) was found in the reference database. Log abundance (total read count of OTU) is plotted on the y-axis. Best match on GenBank is plotted on the x-axis. Blue points represent OTUs discarded by LULU (i.e. OTUs found only in the initial, uncurated OTU tables), and red points represent OTUs retained by the LULU curation, shapes represent species annotations according to best reference database match (GenBank). The “99/100%” clustering level denotes the pure DADA2 approach (100%) and SWARM with a d-value of 3 (99%).

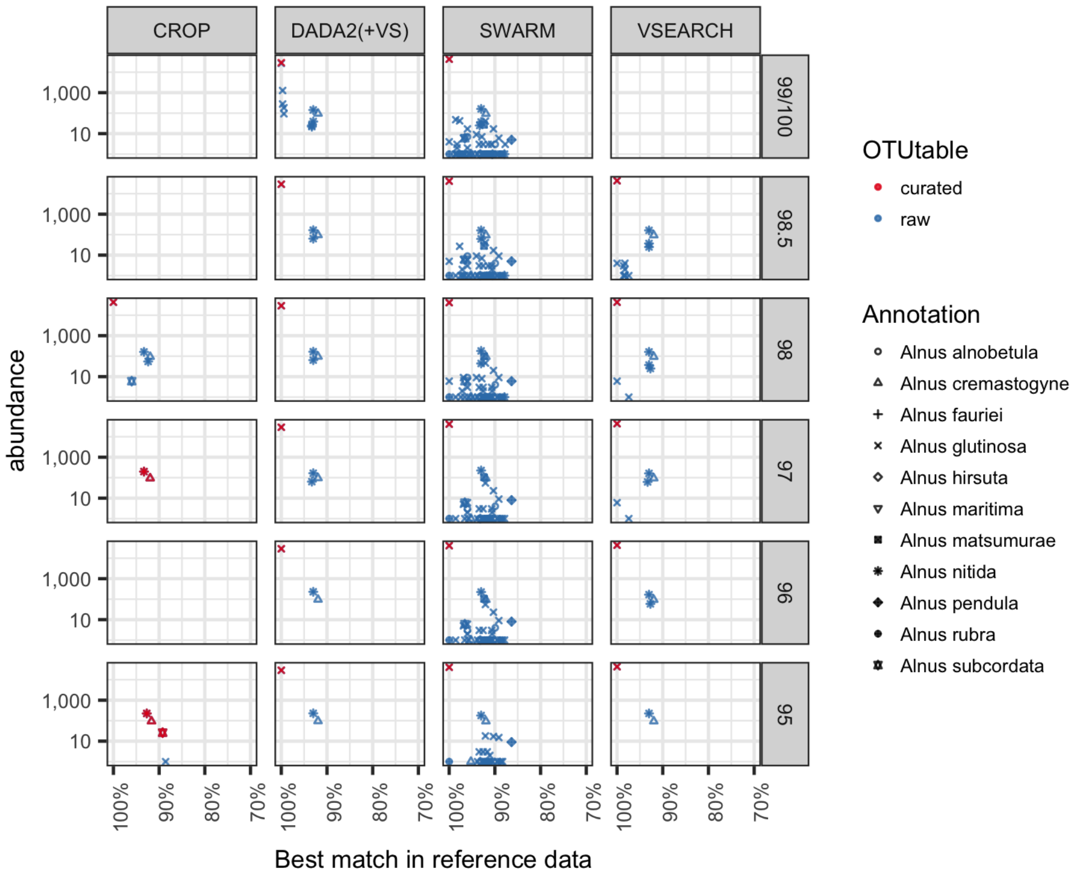

Supplementary Figure 18. Curation effect on OTUs assigned to *Alnus*. One species of Alnus (*A. glutinosa*) was recorded in the plant survey. Curation resulted in accurate diversity measures and correct annotation, except for CROP, where only the 98% approach correlated with expectations from the survey and the other methods. Log abundance (total read count of OTU) is plotted on the y-axis. Best match on GenBank is plotted on the x-axis. Blue points represent OTUs discarded by LULU (i.e. OTUs found only in the initial, uncurated OTU tables), and red points represent OTUs retained by the LULU curation, shapes represent species annotations according to best reference database match (GenBank). The "99/100%" clustering level denotes the pure DADA2 approach (100%) and SWARM with a d-value of 3 (99%).

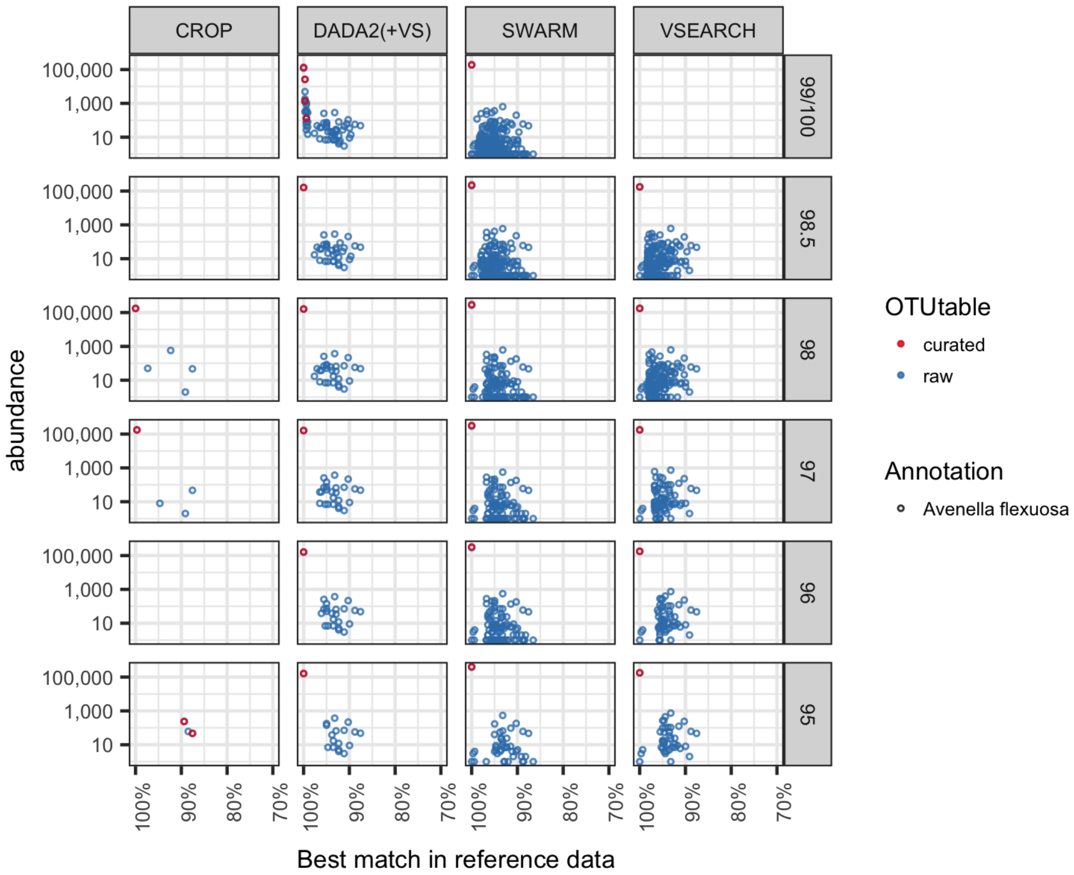

Supplementary Figure 19. Curation effect on OTUs assigned to *Avenella*. The single species of the monotypic genus *Avenella* (*A. flexuosa*) was recorded in the plant survey. Curation resulted in accurate diversity measures and correct annotation, except for 'DADA2-100%' and 'CROP-95%' both finding too many OTUs. Log abundance (total read count of OTU) is plotted on the y-axis. Best match on GenBank is plotted on the x-axis. Blue points represent OTUs discarded by LULU (i.e. OTUs found only in the initial, uncurated OTU tables), and red points represent OTUs retained by the LULU curation, shapes represent species annotations according to best reference database match (GenBank). The “99/100%” clustering level denotes the pure DADA2 approach (100%) and SWARM with a d-value of 3 (99%).

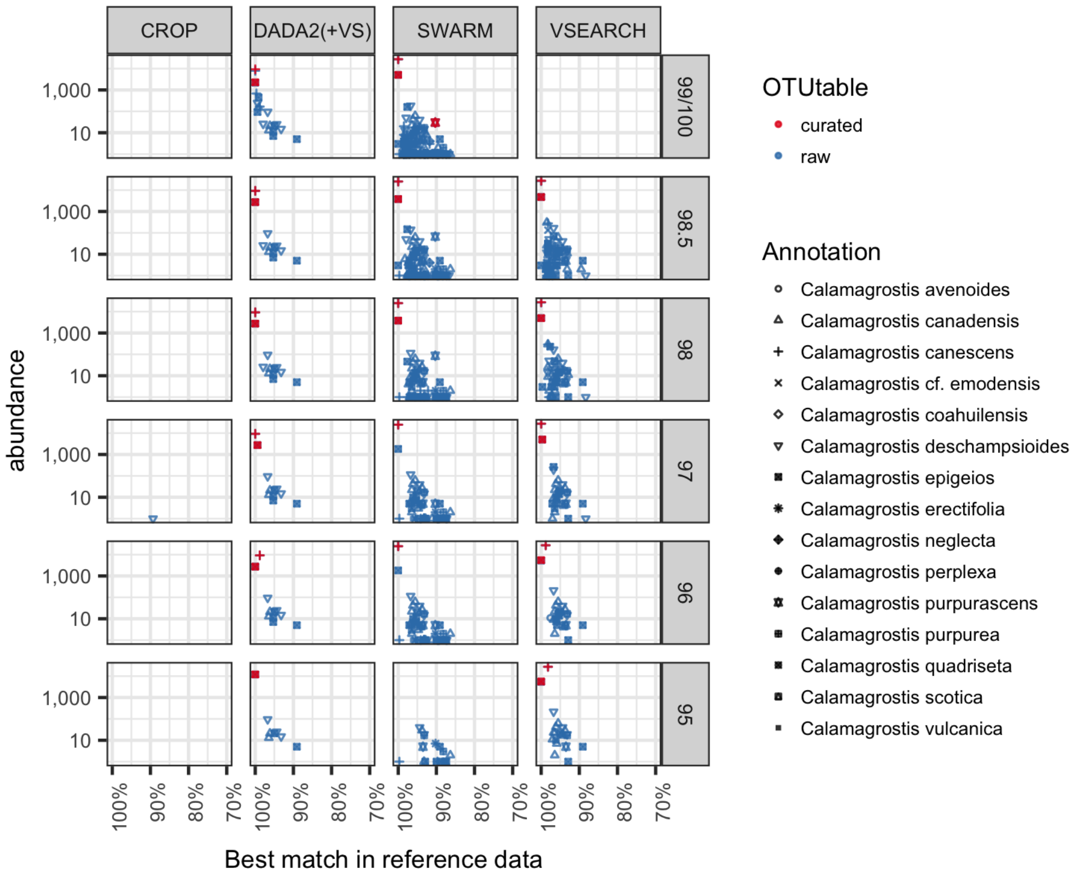

Supplementary Figure 20. Curation effect on OTUs assigned to *Calamagrostis*.

Two species of *Calamagrostis* (*C. canescens* and *C. epigeios*) were recorded in the plant survey. Curation resulted in accurate diversity measures and correct annotation, except for extreme clustering levels and for CROP. In most approaches, far too many OTUs and species names were identified in the un-curated data. Log abundance (total read count of OTU) is plotted on the y-axis. Best match on GenBank is plotted on the x-axis. Blue points represent OTUs discarded by LULU (i.e. OTUs found only in the initial, uncurated OTU tables), and red points represent OTUs retained by the LULU curation, shapes represent species annotations according to best reference database match (GenBank). The “99/100%” clustering level denotes the pure DADA2 approach (100%) and SWARM with a d-value of 3 (99%).

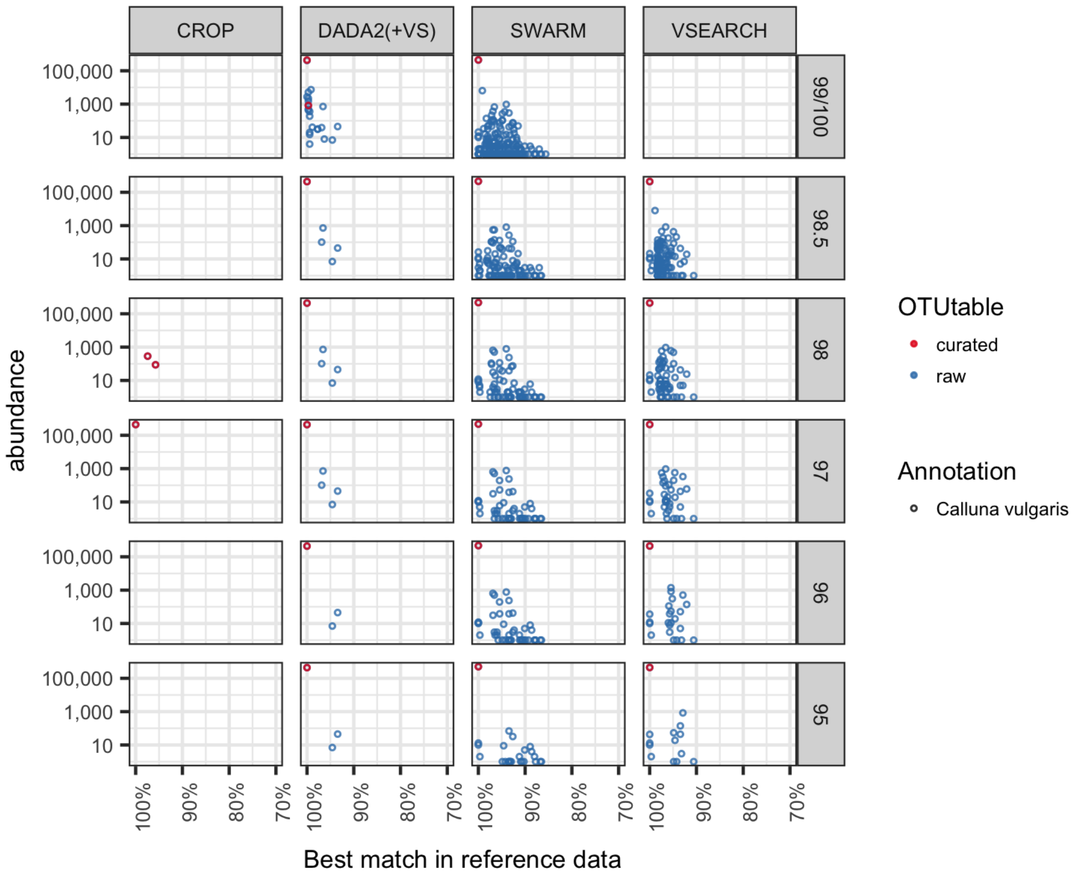

Supplementary Figure 21. Curation effect on OTUs assigned to *Calluna*. The single species of the monotypic genus *Calluna* (*C. vulgaris*) was recorded in the plant survey. Curation resulted in accurate diversity measures and correct annotation, except for 'DADA2-100%', 'CROP-95%' and 'CROP-98%'. In most approaches far too many OTUs were identified in the un-curated data, although they all received the same annotation. Log abundance (total read count of OTU) is plotted on the y-axis. Best match on GenBank is plotted on the x-axis. Blue points represent OTUs discarded by LULU (i.e. OTUs found only in the initial, uncurated OTU tables), and red points represent OTUs retained by the LULU curation, shapes represent species annotations according to best reference database match (GenBank). The “99/100%” clustering level denotes the pure DADA2 approach (100%) and SWARM with a d-value of 3 (99%).

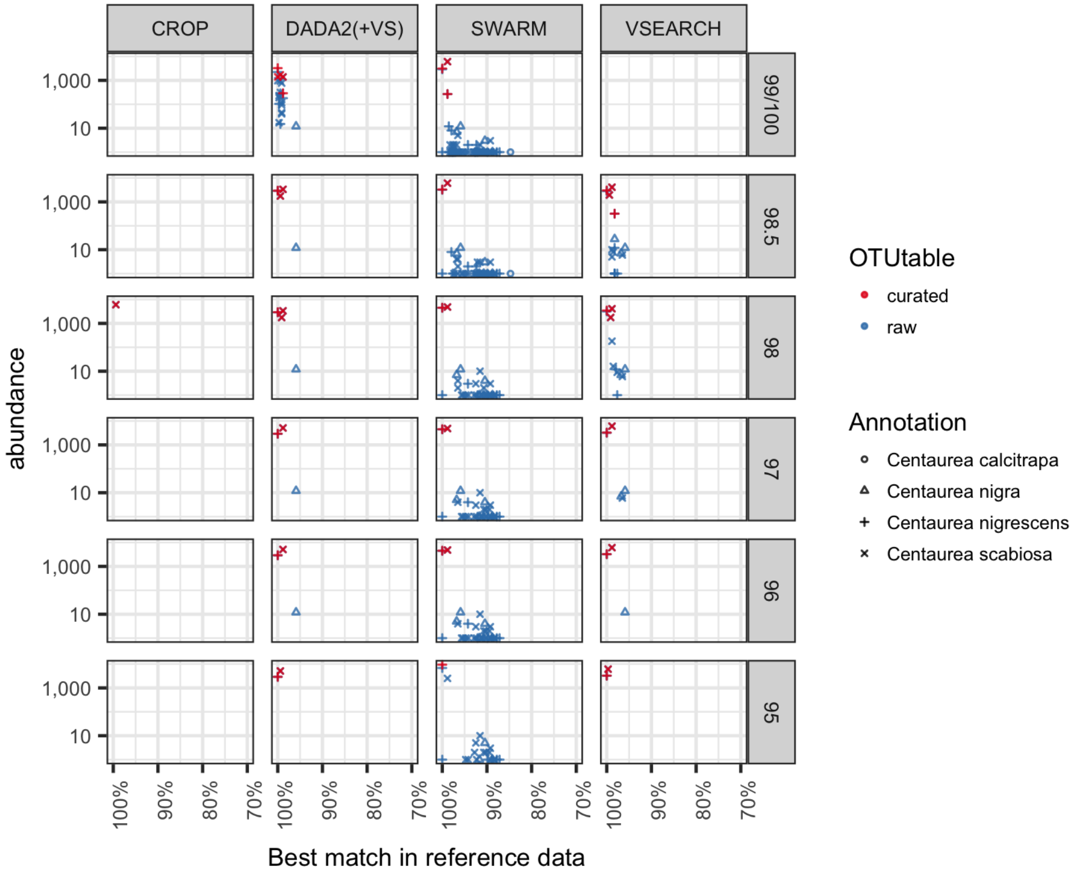

Supplementary Figure 22. Curation effect on OTUs assigned to *Centaurea*. Three species of *Centaurea* (*C. cyanus, C. jacea* and *C. scabiosa*) were recorded in the plant survey. CROP did only identify one species in one approach (98%). Despite suboptimal annotation (only one name match, *C. scabiosa*), the richness estimates were improved by curation. Log abundance (total read count of OTU) is plotted on the y-axis. Best match on GenBank is plotted on the x-axis. Blue points represent OTUs discarded by LULU (i.e. OTUs found only in the initial, uncurated OTU tables), and red points represent OTUs retained by the LULU curation, shapes represent species annotations according to best reference database match (GenBank). The "99/100%" clustering level denotes the pure DADA2 approach (100%) and SWARM with a d-value of 3 (99%).

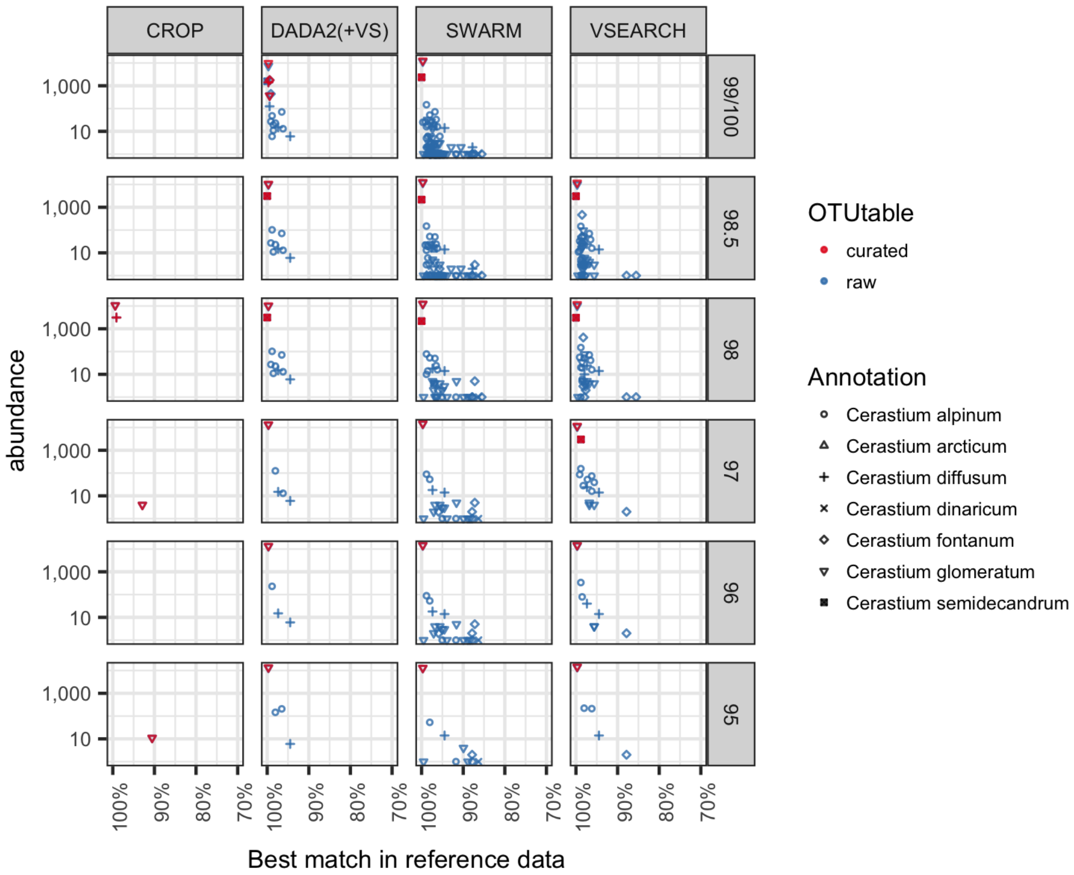

Supplementary Figure 23. Curation effect on OTUs assigned to *Cerastium*. Two taxa of *Cerastium* (*C. fontanum* ssp. *vulgare* var. *vulgare* and *C. semidecandrum*) were recorded in the plant survey. 'CROP 98%' performed better that the two other clustering levels for that method. Curation resulted in better richness estimates for the other approaches, despite deviations in the names between plant survey and OTU for one species. Seemingly, initial clustering levels of 95%, 96% and 97% were too restrictive, as one of the supposed 'good' OTUs was lost. Log abundance (total read count of OTU) is plotted on the y-axis. Best match on GenBank is plotted on the x-axis. Blue points represent OTUs discarded by LULU (i.e. OTUs found only in the initial, uncurated OTU tables), and red points represent OTUs retained by the LULU curation, shapes represent species annotations according to best reference database match (GenBank). The "99/100%" clustering level denotes the pure DADA2 approach (100%) and SWARM with a d-value of 3 (99%).

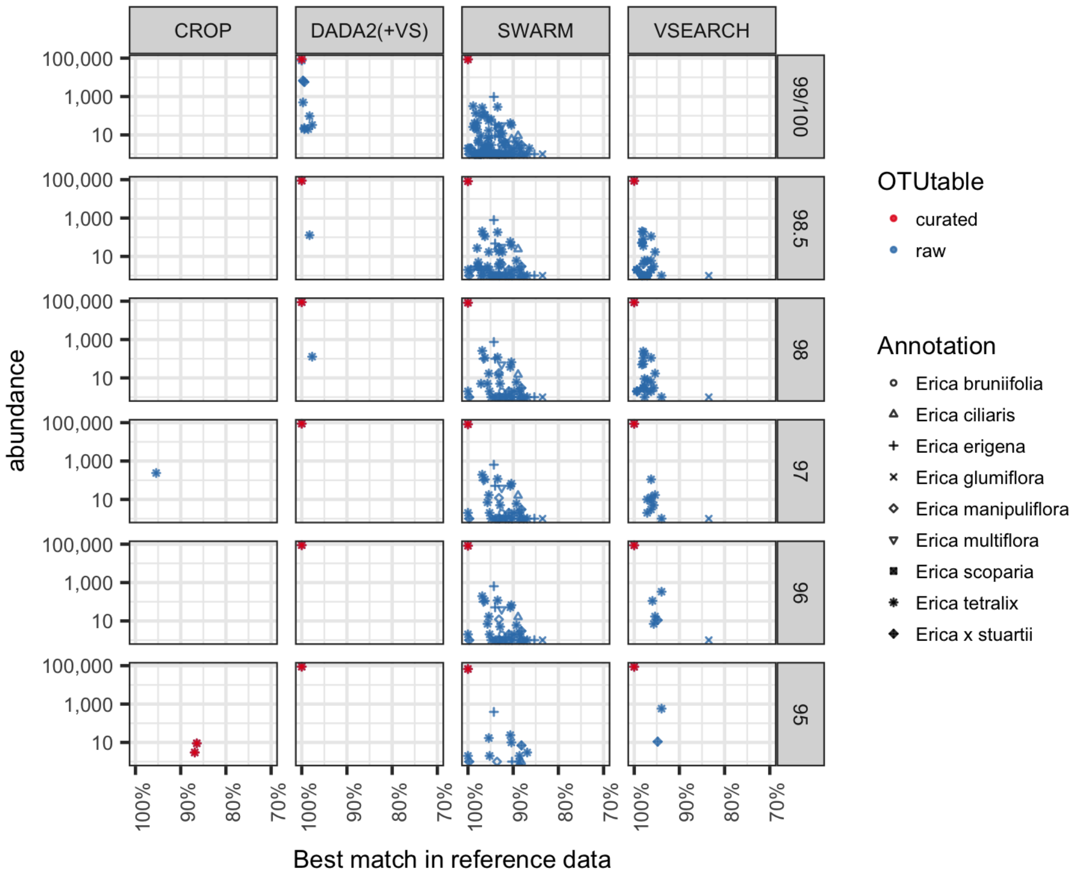

Supplementary Figure 24. Curation effect on OTUs assigned to *Erica*. One species (*E. tetralix*) was recorded in the plant survey. CROP retained suboptimal OTUs with a different abundance and/or number than the other methods. Curation resulted in accurate richness and annotation for the other methods. DADA2+VSEARCH performed accurately without curation for clustering levels 97%, 96% and 95%. Log abundance (total read count of OTU) is plotted on the y-axis. Best match on GenBank is plotted on the x-axis. Blue points represent OTUs discarded by LULU (i.e. OTUs found only in the initial, uncurated OTU tables), and red points represent OTUs retained by the LULU curation, shapes represent species annotations according to best reference database match (GenBank). The “99/100%” clustering level denotes the pure DADA2 approach (100%) and SWARM with a d-value of 3 (99%).

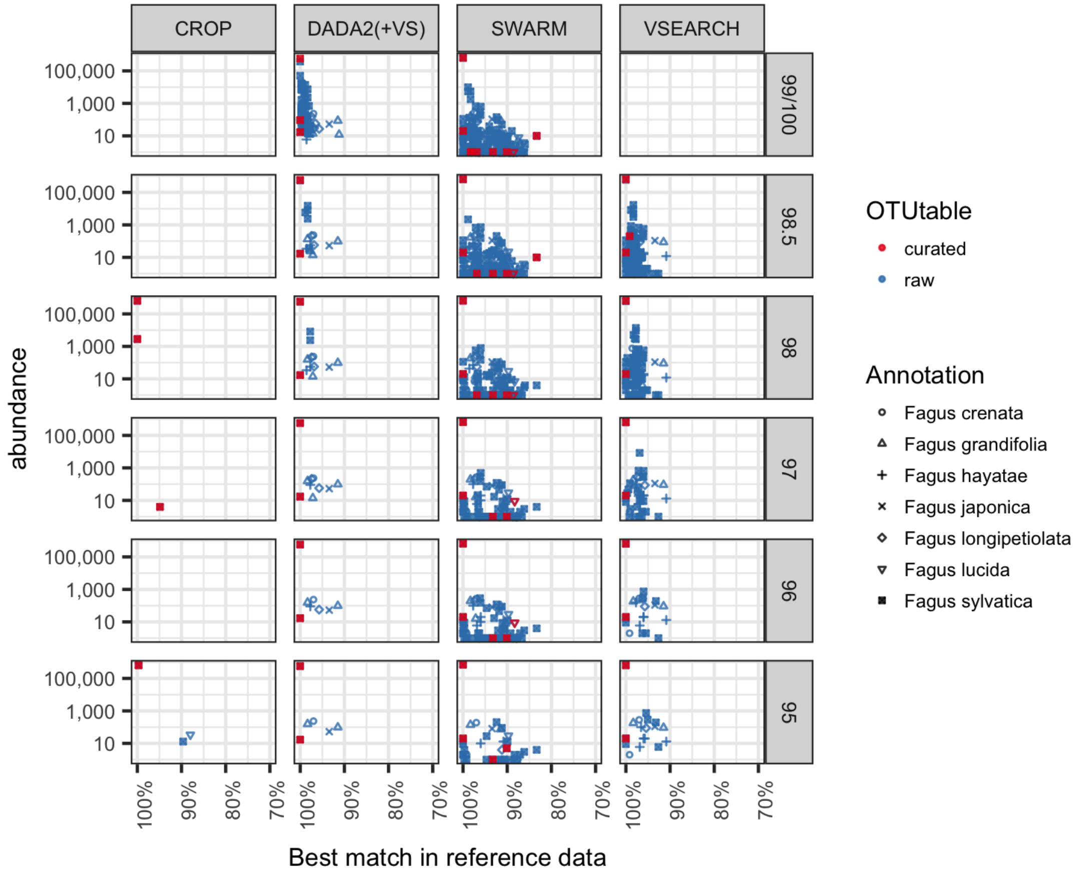

Supplementary Figure 25. Curation effect on OTUs assigned to *Fagus*. One species (*F. sylvatica*) was recorded in the plant survey. CROP initially found the most realistic number of OTUs, despite selecting a suboptimal sequence in the 97% setting, and one OTU impervious to curation in the 98% setting. The single OTU with a 100% match with a read abundance of around 10 (in VSEARCH, DADA2 and SWARM), impervious to curation, was in fact a fungal sequence mathcing a reference in GenBank erroneously annotated as *Fagus*. As this fungal sequence had distribution and abundance pattern contrasting that of Fagus, it was not discarded. Curation made the richness significantly more realistic for all approaches, although the SWARM method retained several redundant OTUs after duration. Log abundance (total read count of OTU) is plotted on the y-axis. Best match on GenBank is plotted on the x-axis. Blue points represent OTUs discarded by LULU (i.e. OTUs found only in the initial, uncurated OTU tables), and red points represent OTUs retained by the LULU curation, shapes represent species annotations according to best reference database match (GenBank). The “99/100%” clustering level denotes the pure DADA2 approach (100%) and SWARM with a d-value of 3 (99%).

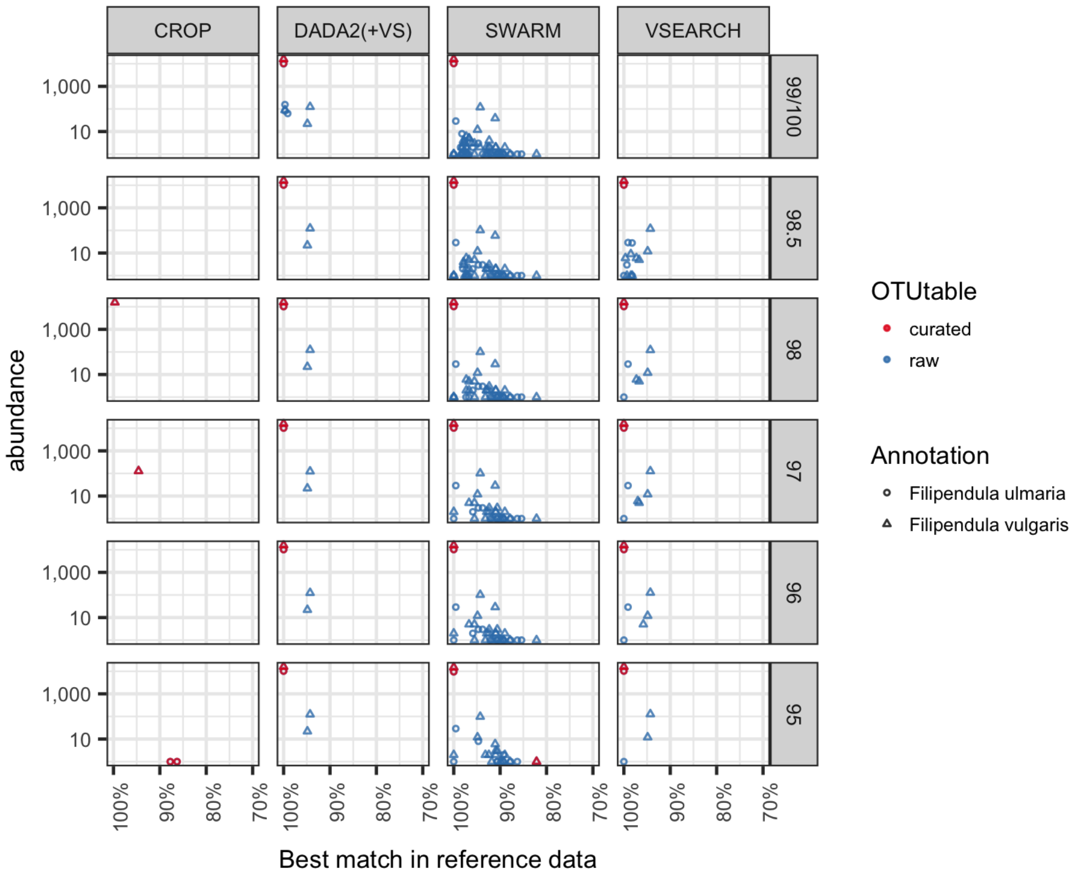

Supplementary Figure 26. Curation effect on OTUs assigned to *Filipendula*. Two species of *Filipendula* (*F. ulmaria* and *F. vulgaris*) were recorded in the plant survey. CROP identified too few and suboptimal OTUs. Curation resulted in accurate taxonomic composition and richness for the other methods, which all identified too many OTUs at all levels. Log abundance (total read count of OTU) is plotted on the y-axis. Best match on GenBank is plotted on the x-axis. Blue points represent OTUs discarded by LULU (i.e. OTUs found only in the initial, uncurated OTU tables), and red points represent OTUs retained by the LULU curation, shapes represent species annotations according to best reference database match (GenBank). The “99/100%” clustering level denotes the pure DADA2 approach (100%) and SWARM with a d-value of 3 (99%).

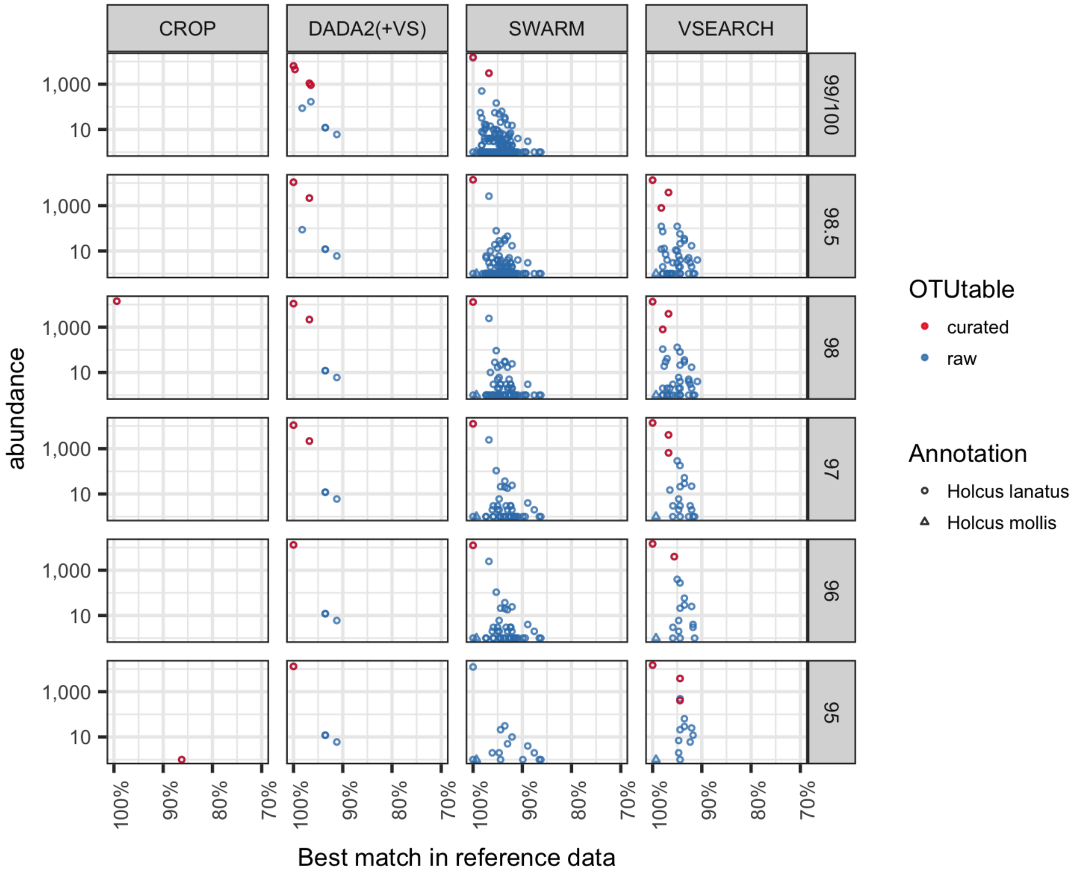

Supplementary Figure 27. Curation effect on OTUs assigned to *Holcus*. Curation effect on all OTUs assigned to the genus *Holcus*. Two species (*H. lanatus* and *H. mollis*) were recorded in the plant survey. CROP identified too few and suboptimal OTUs. Curation resulted in accurate richness estimates for the other methods at clustering levels of 97% and upwards. Both retained OTUs were assigned to *H. lanatus*, one perfectly and the other with lower match, probably due to H. mollis not being represented in the reference database. Log abundance (total read count of OTU) is plotted on the y-axis. Best match on GenBank is plotted on the x-axis. Blue points represent OTUs discarded by LULU (i.e. OTUs found only in the initial, uncurated OTU tables), and red points represent OTUs retained by the LULU curation, shapes represent species annotations according to best reference database match (GenBank). The “99/100%” clustering level denotes the pure DADA2 approach (100%) and SWARM with a d-value of 3 (99%).

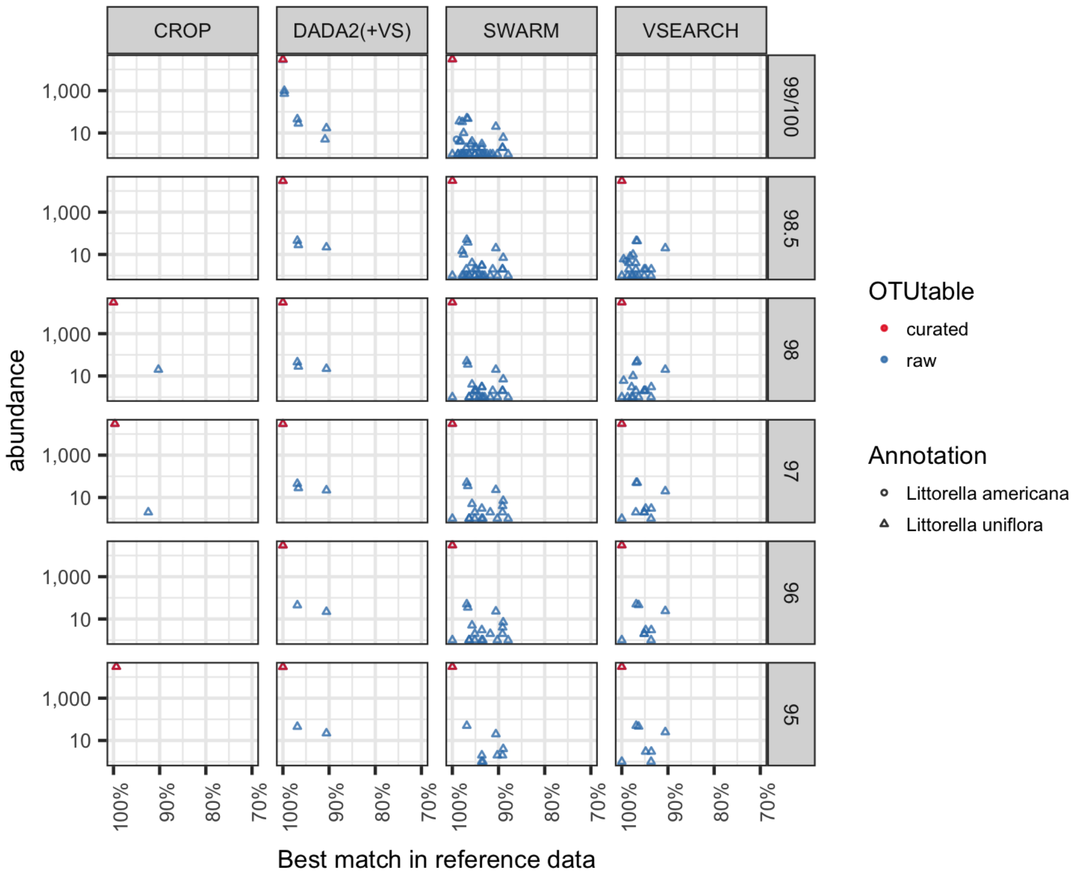

Supplementary Figure 28. Curation effect on OTUs assigned to *Littorella*. Curation effect on all OTUs assigned to the genus *Littorella*, which is sometimes considered a part of *Plantago* (see below for data on *Plantago*). One species (*L. uniflora*) is known from the study area and was recorded in the plant survey. All approaches identified the correctly annotated species, but only 'CROP-95%' did not identify any extra OTUs. The other approaches initially identified too many OTUs at all clustering levels. Curation resulted in accurate richness for all approaches. Log abundance (total read count of OTU) is plotted on the y-axis. Best match on GenBank is plotted on the x-axis. Blue points represent OTUs discarded by LULU (i.e. OTUs found only in the initial, uncurated OTU tables), and red points represent OTUs retained by the LULU curation, shapes represent species annotations according to best reference database match (GenBank). The “99/100%” clustering level denotes the pure DADA2 approach (100%) and SWARM with a d-value of 3 (99%).

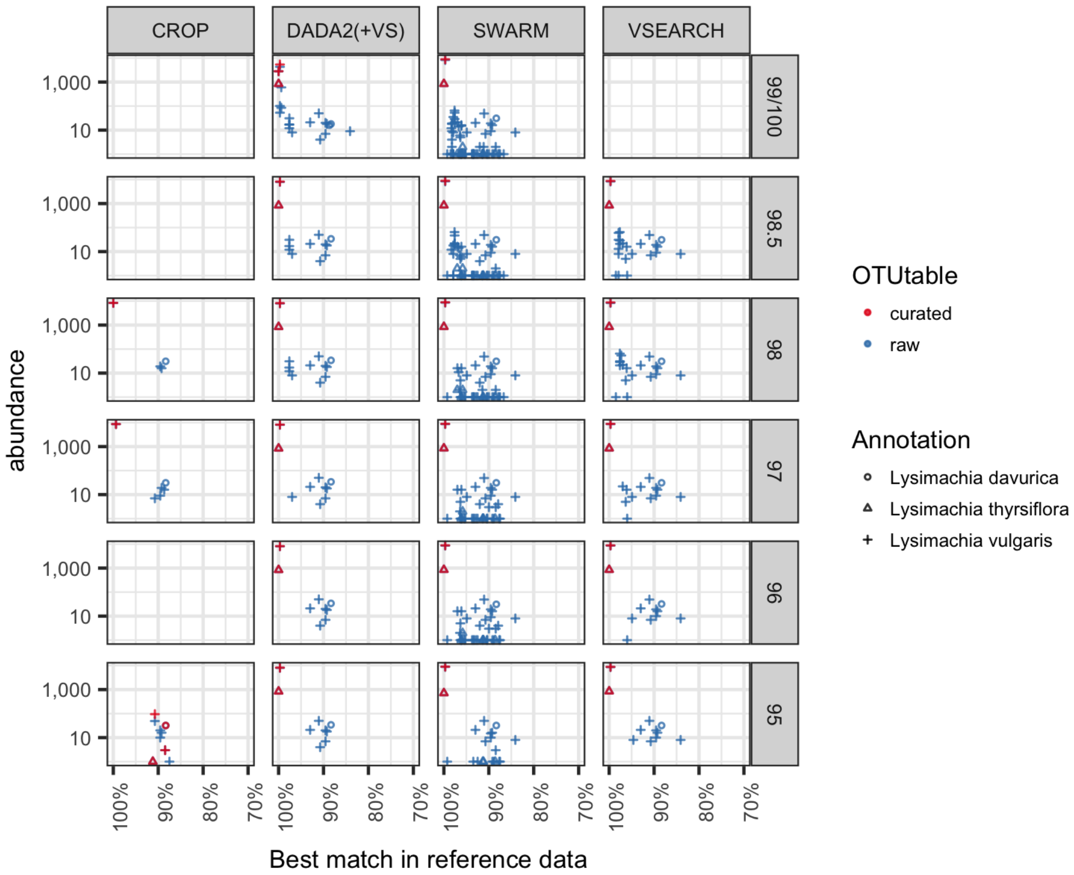

Supplementary Figure 29. Curation effect on OTUs assigned to *Lysimachia*. Two species (*L. thyrsiflora* and *L. vulgaris*) were recorded in the plant survey. Except for CROP, curation resulted in accurate taxonomic composition and richness for all methods, which all initially identified too many OTUs at all levels. Log abundance (total read count of OTU) is plotted on the y-axis. Best match on GenBank is plotted on the x-axis. Blue points represent OTUs discarded by LULU (i.e. OTUs found only in the initial, uncurated OTU tables), and red points represent OTUs retained by the LULU curation, shapes represent species annotations according to best reference database match (GenBank). The "99/100%" clustering level denotes the pure DADA2 approach (100%) and SWARM with a d-value of 3 (99%).

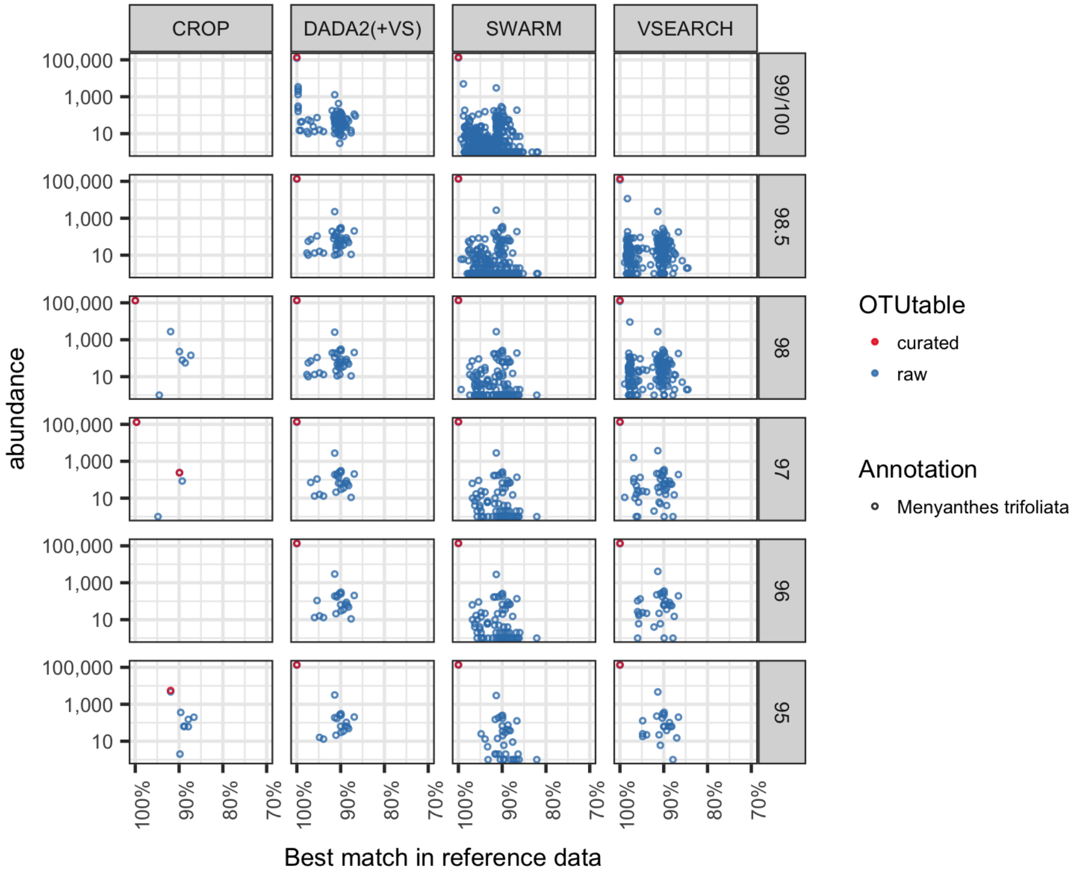

Supplementary Figure 30. Curation effect on OTUs assigned to *Menyanthes*.

The single species of the monotypic genus *Menyanthes* (*M. trifoliata*) was recorded in the plant survey. All methods initially identified too many OTUs, despite correct annotation. Curation resulted in accurate richness for all methods except 'CROP-97%' with one OTU too much. Log abundance (total read count of OTU) is plotted on the y-axis. Best match on GenBank is plotted on the x-axis. Blue points represent OTUs discarded by LULU (i.e. OTUs found only in the initial, uncurated OTU tables), and red points represent OTUs retained by the LULU curation, shapes represent species annotations according to best reference database match (GenBank). The “99/100%” clustering level denotes the pure DADA2 approach (100%) and SWARM with a d-value of 3 (99%).

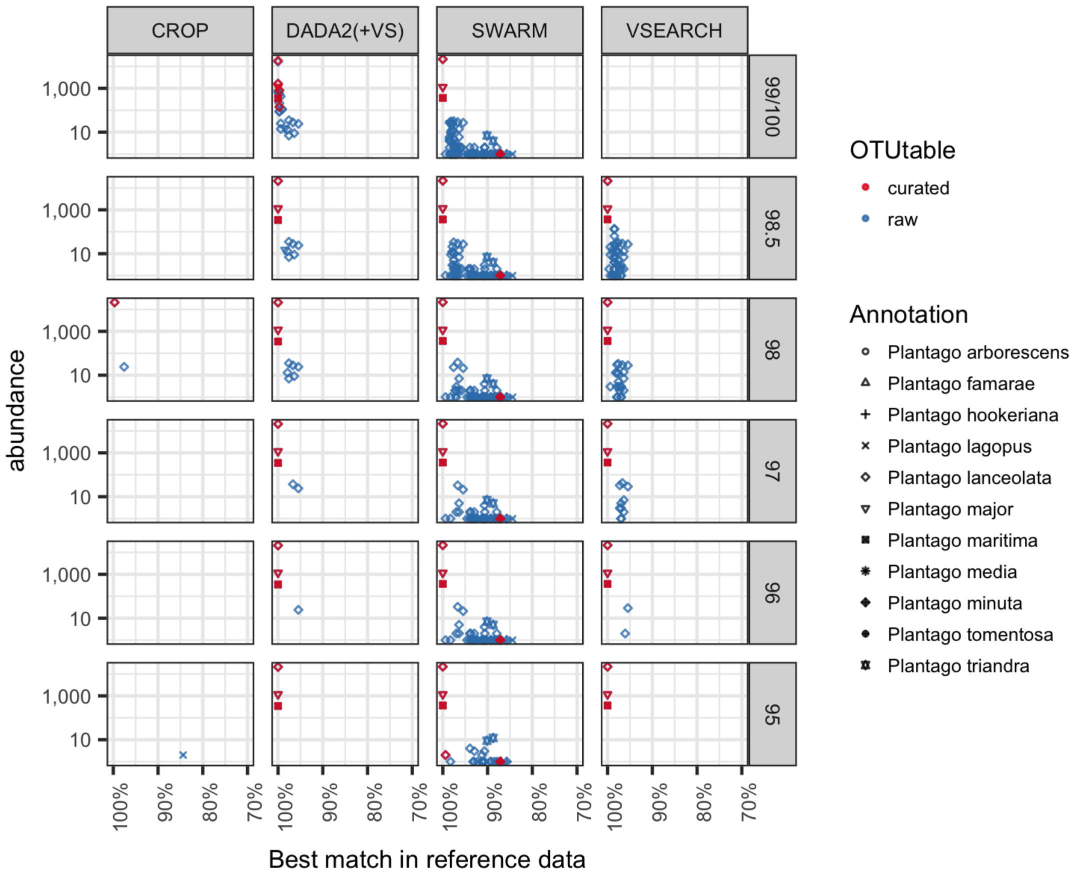

Supplementary Figure 31. Curation effect on OTUs assigned to *Plantago*. Three species (*P. lanceolata, P. major* and *P. maritima*) were recorded in the plant survey (for data on *Littorella uniflora* (=*P. uniflora*) see above). CROP selected suboptimal OTUs and/or too few. Curation resulted in accurate richness and annotation for all other methods. DADA2-95% and VSEACRH-95% performed accurate without curation also. Log abundance (total read count of OTU) is plotted on the y-axis. Best match on GenBank is plotted on the x-axis. Blue points represent OTUs discarded by LULU (i.e. OTUs found only in the initial, uncurated OTU tables), and red points represent OTUs retained by the LULU curation, shapes represent species annotations according to best reference database match (GenBank). The “99/100%” clustering level denotes the pure DADA2 approach (100%) and SWARM with a d-value of 3 (99%).

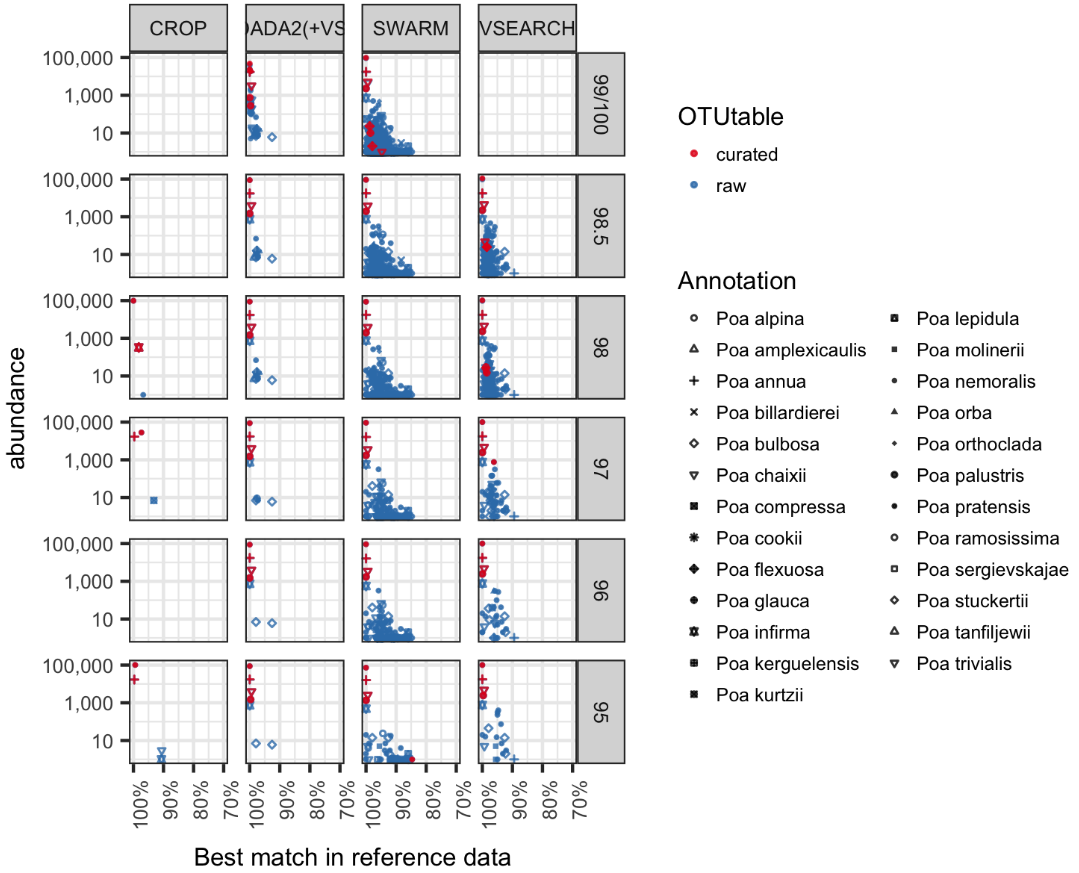

Supplementary Figure 32. Curation effect on OTUs assigned to *Poa*. Curation effect on all OTUs assigned to the genus *Poa*. Five species of *Poa* (*P. annua, P. compressa, P. nemoralis, P. pratensis* and *P. trivialis*) were recorded in the plant survey. Grasses often pose problems in molecular identification. Here, curation resulted in a very good correspondence between what could be expected from the plant survey, despite richness being slightly underestimated (4 out of five 5 species) in most approaches and one or two ill-assigned species. Log abundance (total read count of OTU) is plotted on the y-axis. Best match on GenBank is plotted on the x-axis. Blue points represent OTUs discarded by LULU (i.e. OTUs found only in the initial, uncurated OTU tables), and red points represent OTUs retained by the LULU curation, shapes represent species annotations according to best reference database match (GenBank). The “99/100%” clustering level denotes the pure DADA2 approach (100%) and SWARM with a d-value of 3 (99%).

Supplementary tables

Method	Level	Binary	Abundance
**CROP**	98%	0.99	1
**CROP**	97%	0.993	1
**CROP**	95%	0.986	1
**DADA2(+VS)**	99/100%	0.879	0.988
**DADA2(+VS)**	98.50%	0.944	0.997
**DADA2(+VS)**	98%	0.969	0.998
**DADA2(+VS)**	97%	0.976	0.999
**DADA2(+VS)**	96%	0.979	0.999
**DADA2(+VS)**	95%	0.981	0.999
**SWARM**	99/100%	0.764	0.987
**SWARM**	98.50%	0.811	0.993
**SWARM**	98%	0.85	0.996
**SWARM**	97%	0.857	0.996
**SWARM**	96%	0.86	0.996
**SWARM**	95%	0.903	0.998
**VSEARCH**	98.50%	0.793	0.988
**VSEARCH**	98%	0.761	0.992
**VSEARCH**	97%	0.906	0.997
**VSEARCH**	96%	0.93	0.998
**VSEARCH**	95%	0.944	0.999

Supplementary Table 1. Effect of curation on community dissimilarity measures. The effect of curation of community dissimilarity measures was evaluated by comparing Bray-Curtis dissimilarity measures based of pairs of un-curated vs. curated OTU tables with the Mantel test. Comparisons were done for both binary OTU tables and tables with abundance (read count) information using a hellinger transformation. Mantel r-statistics are show for both approaches. All p-values were below 0.001. The high correspondence of un-curated and curated tables, show that curation has little effect on community dissimilarity measures. The highest curation effect (lowest correspondence) was seen binary tables produced with SWARM and VSEARCH.

Method	Level	Binary	Abundance
**CROP**	98%	0.75/0.76	0.63/0.63
**CROP**	97%	0.7/0.71	0.57/0.57
**CROP**	95%	0.66/0.67	0.57/0.58
**DADA2(+VS)**	99/100%	0.66/0.73	0.58/0.6
**DADA2(+VS)**	98.50%	0.73/0.76	0.62/0.63
**DADA2(+VS)**	98%	0.74/0.76	0.63/0.63
**DADA2(+VS)**	97%	0.76/0.77	0.63/0.64
**DADA2(+VS)**	96%	0.76/0.77	0.64/0.64
**DADA2(+VS)**	95%	0.76/0.77	0.64/0.64
**SWARM**	99/100%	0.64/0.76	0.63/0.66
**SWARM**	98.50%	0.67/0.76	0.65/0.67
**SWARM**	98%	0.68/0.75	0.65/0.66
**SWARM**	97%	0.67/0.73	0.66/0.66
**SWARM**	96%	0.67/0.73	0.66/0.66
**SWARM**	95%	0.66/0.72	0.66/0.67
**VSEARCH**	98.50%	0.64/0.77	0.62/0.65
**VSEARCH**	98%	0.63/0.76	0.63/0.65
**VSEARCH**	97%	0.73/0.78	0.64/0.65
**VSEARCH**	96%	0.75/0.78	0.65/0.66
**VSEARCH**	95%	0.76/0.78	0.65/0.66
**BDOTU3 (one step)**	a=10	0.65	0.61
**DBOTU3 (one step)**	a=10	0.68	0.62

Supplementary Table 2. Effect of curation on correspondence with plant community dissimilarity measures. Values before slashes are Mantel r-statistic of OTU based dissimilarity vs. plant dissimilarity before curation, and values after slashes are Mantel r-statistic of OTU based dissimilarity vs. plant dissimilarity after curation, all p-values < 0.001. All dissimilarity measures are based on Bray-Curtis distances. For the OTU tables, dissimilarity measures were calculated both for binary data and for abundance (read count) data after hellinger transformation. The two last rows show values for dbotu3 used as a 'one-step' clustering algorithm as intended, and does not have a 'curated value'. The results show that all OTU tables show good correspondence with plant community data. The best correlation is seen for binary data. The effect of curation is small to slightly positive - highest for binary tables produced with SWARM and VSEARCH.

Method	Level	Correlation (R^2)	Slope	Intercept
**CROP**	98%	0.56/0.58/0.57/**0.59/0.59**	**0.32/0.31/0.3/0.31/0.3**	3.8/3.2/3.3/**2.9/2.9**
**CROP**	97%	0.54/0.53/0.58/0.59/**0.6**	**0.24/0.23/0.23/0.23/0.23**	2/2/1.6/1.6/**1.4**
**CROP**	95%	0.48/0.54/0.57/**0.6/0.6**	**0.24/0.21/0.22/0.22/0.22**	1.8/1.8/1.4/1.3/**1.1**
**DADA2(+VS)**	99/100%	0.42/0.42/0.43/0.45/**0.56**	**0.77/0.77/0.6/0.56/0.53**	15.6/15.5/12.1/10.5/**3.6**
**DADA2(+VS)**	98.50%	0.54/0.54/0.59/0.6/**0.63**	**0.55/0.55/0.46/0.46/0.44**	6.4/6.4/4.6/4.1/**1.8**
**DADA2(+VS)**	98%	0.55/0.55/0.61/0.62/**0.64**	**0.52/0.52/0.44/0.44/0.42**	6/6/4.2/3.6/**1.9**
**DADA2(+VS)**	97%	0.57/0.57/0.62/0.62/**0.65**	**0.49/0.49/0.43/0.43/0.42**	5/5/3.5/3/**1.8**
**DADA2(+VS)**	96%	0.62/0.62/0.66/0.66/**0.67**	**0.47/0.47/0.42/0.42/0.41**	4/4/2.8/2.5/**1.3**
**DADA2(+VS)**	95%	0.61/0.61/0.66/0.66/**0.68**	**0.44/0.44/0.41/0.41/0.41**	3.7/3.7/2.5/2.3/**1.1**
**SWARM**	99/100%	0.15/0.17/0.32/0.46/**0.64**	3.49/1.5/1.36/**0.99/0.64**	49.6/32.9/18.8/12/**2.1**
**SWARM**	98.50%	0.2/0.24/0.36/0.48/**0.67**	2.35/1.15/1.22/**0.91/0.62**	26.4/21.2/12.2/9.8/**1.8**
**SWARM**	98%	0.25/0.29/0.37/0.49/**0.69**	1.81/0.99/1.13/**0.87/0.58**	18.1/15.6/10/8.6/**2.1**
**SWARM**	97%	0.27/0.32/0.4/0.51/**0.69**	1.55/0.87/1.05/**0.82/0.56**	14.7/13.9/9/8.6/**2.8**
**SWARM**	96%	0.27/0.33/0.4/0.51/**0.7**	1.55/**0.88/1.05/0.82/0.56**	14.1/13.5/8.7/8.4/**2.8**
**SWARM**	95%	0.39/0.5/0.45/0.56/**0.71**	1.15/**0.74/0.94/0.77/0.53**	4.5/5.9/3.4/4.6/**2.3**
**VSEARCH**	98.50%	0.15/0.17/0.34/0.44/**0.63**	2.15/1.62/1.01/**0.87/0.73**	62.7/43.6/20.4/13.1/**1.6**
**VSEARCH**	98%	0.17/0.2/0.36/0.45/**0.59**	1.58/1.28/**0.95/0.83/0.7**	41.5/32.1/20.4/13.1/**1.7**
**VSEARCH**	97%	0.22/0.27/0.36/0.45/**0.64**	**0.92/0.83/0.72/0.67/0.61**	22/17.1/13.2/9.6/**1.8**
**VSEARCH**	96%	0.27/0.32/0.41/0.48/**0.64**	**0.8/0.74/0.68/0.65/0.57**	16.4/12.9/10.1/8.1/**1.9**
**VSEARCH**	95%	0.34/0.39/0.48/0.53/**0.66**	**0.7/0.65/0.63/0.63/0.55**	12.3/10/8/6.4/**1.9**
**DBOTU3 (one-step)**	a=10	0.08	1.04	107.1
**DBOTU3 (one-step)**	a=0	0.18	1.04	59.7

Supplementary Table 3. Richness correspondence – benchmarking of dbotu3 (for curation), singleton culling and 'one-step' clustering with dbotu3. Effect of curation on richness correspondence by singleton culling and post clustering curation with dbotu3 compared to curation with LULU. R2 denotes the coefficient of determination of the linear regression of OTU count vs. plant richness, slope and intercept denotes the constants of the inferred linear regression. Values on the first position are values for the initial (un-curated) OTU tables, values in the second position are derived from OTU tables where singletons have been removed, values in the third position are derived from tables curated with dbotu3 using an abundance criterion of 10, values in the fourth position are derived from tables curated with dbotu3 using an abundance criterion of 0, and values in the last position are derived from tables curated with LULU. The two last rows show values for dbotu3 used as a 'one-step' clustering algorithm as intended, and does not have values for singleton culling or curation. The best value or all values satisfying our criteria for correspondence are indicated in bold for each approach (method+clustering level). For all approaches LULU curation results in the best metrics for correlation and intercept. The slope is below 1 for all LULU curated tables, satisfying our criterium for good correspondence. This is also seen for dbotu3 (with a=0) and several other approaches.

Method	Level	Total_OTUs	Taxonomic redundancy	Average best match	Betadiversity
**CROP**	98%	**369/328/254/235/241**	28%/24%/11%/8%/**7%**	95.8%/96.2%/97.5%/**97.8%**/97.5%	25.9/**24.5/19.4/18.2/19.1**
**CROP**	97%	**249/229/169/162/174**	22%/21%/8%/6%/**6%**	94.7%/95%/**96.8%/96.8%**/96.4%	25/**24.4/18.6/17.9/19.5**
**CROP**	95%	**383/285/253/214/252**	28%/21%/11%/7%/**8%**	92.2%/93.3%/93.7%/**94.5%**/93.7%	39/33.3/29.2/25.2/29.9
**DADA2(+VS)**	100%	2568/2561/1229/830/761	77%/77%/64%/53%/**45%**	97.7%/97.8%/98.9%/**99%**/98.8%	62.8/62.7/38.9/28.9/36.3
**DADA2(+VS)**	98.50%	1141/1141/**557/455/430**	53%/53%/27%/19%/**13%**	96.7%/96.7%/98.2%/98.5%/**98.7%**	46.9/46.9/28.3/**23.9**/26.5
**DADA2(+VS)**	98%	1033/1033/**516/428/402**	50%/50%/23%/17%/**10%**	96.6%/96.6%/98.2%/98.5%/**98.7%**	45.2/45.2/27.7/**23.7**/25.5
**DADA2(+VS)**	97%	842/842/**447/385/365**	43%/43%/17%/12%/**7%**	96.4%/96.4%/98.1%/98.5%/**98.6%**	40.4/40.4/25.5/**22.5/23.**7
**DADA2(+VS)**	96%	721/721/**410/360/341**	37%/37%/14%/9%/**6%**	96.2%/96.2%/98%/98.4%/**98.6%**	37.3/37.3/**24.7/22/22.9**
**DADA2(+VS)**	95%	622/622/**384/339/324**	32%/32%/12%/8%/**5%**	96.2%/96.2%/98%/98.4%/**98.5%**	34.2/34.2/**23.9/21.5/22.3**
**SWARM**	99%	14828/5870/1934/839/**520**	93%/86%/70%/43%/**18%**	95.1%/95.9%/96.6%/97.1%/**97.9%**	90.5/71.6/30.6/**18.9/22.5**
**SWARM**	98.50%	8422/3775/1704/792/**467**	88%/80%/65%/41%/**13%**	94.2%/95.2%/95.6%/96.7%/**97.8%**	81.5/64/32.6/**20/21.2**
**SWARM**	98%	5779/2783/1584/759/**430**	84%/74%/63%/40%/**9%**	93.6%/94.7%/94.8%/96.3%/**97.7%**	74.8/58.1/33.8/**20.6/20.6**
**SWARM**	97%	4585/2274/1465/729/4**01**	81%/70%/62%/40%/**8%**	93.3%/94.5%/94.4%/96%/**97.7%**	70/53.6/33.8/**20.6/19.1**
**SWARM**	96%	4547/2257/1464/727/**401**	81%/70%/62%/40%/**8%**	93.2%/94.4%/94.4%/96%/**97.7%**	70/53.6/34/**20.6/19.1**
**SWARM**	95%	2500/1348/1286/671/**362**	70%/57%/59%/38%/**9%**	92.6%/93.8%/93.7%/95.7%/**97.3%**	59.4/44.7/37.7/**22.6/18.5**
**VSEARCH**	98.50%	8008/6001/1581/803/**558**	90%/88%/66%/44%/**23%**	97.4%/97.4%/97.7%/98.2%/**98.4%**	60.2/62.1/29.5/**19.2/21.9**
**VSEARCH**	98%	4815/3939/1336/761/**517**	85%/83%/61%/42%/**20%**	96.8%/96.9%/97.6%/98.1%/**98.4%**	51.6/53.1/25.9/**18.8/20.9**
**VSEARCH**	97%	2425/2063/989/642/**458**	72%/69%/49%/32%/**13%**	96.1%/96.2%/97.2%/97.8%/**98.4%**	46.5/46.5/26.9/**20.3/21**
**VSEARCH**	96%	1740/1505/790/582/**415**	64%/61%/41%/28%/**10%**	95.7%/95.9%/97.1%/97.7%/**98.3%**	40.9/40.7/**24.4/19.9/20.1**
**VSEARCH**	95%	1320/1161/694/**538/396**	56%/53%/36%/25%/**9%**	95.5%/95.6%/97%/97.5%/**98.2%**	37.5/37.1/**24.2/20/19.8**
**DBOTU (one-step)**	a=10	2267	78%	**99.0%**	**16.0**
**DBOTU (one-step)**	a=0	1136	65%	**99.0%**	**12.1**

Supplementary Table 4. Supplementary metrics – benchmarking of dbotu3 (for curation), singleton culling and 'one-step' clustering with dbotu3. Effect of curation on total OTU count, taxonomic redundancy, average best reference database match, and betadiversity. Total OTUs is the count of total unique OTUs for each method, taxonomic redundancy is calculated as the proportion of OTUs with a redundant taxonomic assignment, avg. best match is the average of the best GenBank match for all OTUs for each method, and β-diversity is the average α-diversity divided by γ-diversity. Values on the first position are values for the initial (un-curated) OTU tables, values in the second position are derived from OTU tables where singletons have been removed, values in the third position are derived from tables curated with dbotu3 using an abundance criterion of 10, values in the fourth position are derived from tables curated with dbotu3 using an abundance criterion of 0, and values in the last position are derived from tables curated with LULU. The two last rows show values for dbotu3 used as a 'one-step' clustering algorithm as intended, and does not have values for singleton culling or curation The best value or values or all values satisfying our criteria for correspondence are indicated in bold for each approach (method+clustering level). For total OTUs all values below 564 have been indicated. For betadiversity, values below 25 have been indicated to reflect the more relaxed the criterium is approaching the betadiversity of 17.3 for the plant data. Generally, LULU curation is the approach resulting in the best metrics. The criteria for total OTU count and betadiversity are also satisfied by other treatments for the CROP algorithm and DADA2.

Method	Level	OTU Read count	Plant OTU read count	Plant OTU read count excluding singletons
**CROP**	98%	4130397	3714574	3714461
**CROP**	97%	3326185	2817352	2817281
**CROP**	95%	3215049	2642251	2642085
**DADA2(+VS)**	100%	5725783	5052066	5052058
**DADA2(+VS)**	98.50%	5709598	5053333	5053333
**DADA2(+VS)**	98%	5709610	5053345	5053345
**DADA2(+VS)**	97%	5709759	5058996	5058996
**DADA2(+VS)**	96%	5709856	5058974	5058974
**DADA2(+VS)**	95%	5710036	5059610	5059610
**SWARM**	99%	6315687	5603133	5592489
**SWARM**	98.50%	6315687	5603399	5597635
**SWARM**	98%	6315687	5604972	5601149
**SWARM**	97%	6315687	5604914	5601910
**SWARM**	96%	6315687	5604920	5601951
**SWARM**	95%	6315687	5609876	5608317
**VSEARCH**	98.50%	6262251	5555568	5550823
**VSEARCH**	98%	6269817	5562362	5559870
**VSEARCH**	97%	6281916	5574137	5573126
**VSEARCH**	96%	6289642	5582255	5581540
**VSEARCH**	95%	6294320	5591296	5590789
**DBOTU3**	a=0	6624089	5607927	-
**DBOTU3**	a=10	6624089	5604744	-

Supplementary Table 5. Number of reads per approach. Number of reads in the OTU tables after OTU clustering, after selecting only plant OTUs, and after culling of singletons. The two last rows show values for dbotu3 used as a 'one-step' clustering algorithm as intended, and does not have values for singleton culling.

	100%	99%	98.5%	98%	97%	96%	95%
**DADA2**	co clustering						
**VSEARCH**			98.5	98	97	96	95
**DADA2 + VSEARCH**			98.5	98	97	96	95
**CROP**				l=0.5, u=1.0	l=1, u=1.5		l=1.5, u=2.5
**SWARM**		d=3	d=5	d=7	d=10	d=13	d=15

Supplementary Table 6. Algorithms and clustering levels. In the study we used the following algorithms and levels for clustering of sequences into OTUs. The clustering corresponding levels (d-values) for SWARM was calculated from an average amplicon length of 340 nucleotides (except for the 95% level where we used 15, instead of the implied 17), and as SWARM can absorb linked errors we also tested d=3 (≈99%). We only tested three levels for CROP as it is a time-consuming algorithm.
